# Nanostructured Antimicrobial Peptides: Crucial Steps of Overcoming the Bottleneck for Clinics

**DOI:** 10.3389/fmicb.2021.710199

**Published:** 2021-08-12

**Authors:** Zhanyi Yang, Shiqi He, Hua Wu, Ting Yin, Lili Wang, Anshan Shan

**Affiliations:** Institute of Animal Nutrition, Northeast Agricultural University, Harbin, China

**Keywords:** antimicrobial peptide, inorganic nanoparticles, organic nanoparticles, self-assembly, controlled drug release

## Abstract

The security issue of human health is faced with dispiriting threats from multidrug-resistant bacteria infections induced by the abuse and misuse of antibiotics. Over decades, the antimicrobial peptides (AMPs) hold great promise as a viable alternative to treatment with antibiotics due to their peculiar antimicrobial mechanisms of action, broad-spectrum antimicrobial activity, lower drug residue, and ease of synthesis and modification. However, they universally express a series of disadvantages that hinder their potential application in the biomedical field (e.g., low bioavailability, poor protease resistance, and high cytotoxicity) and extremely waste the abundant resources of AMP database discovered over the decades. For all these reasons, the nanostructured antimicrobial peptides (Ns-AMPs), based on a variety of nanosystem modification, have made up for the deficiencies and pushed the development of novel AMP-based antimicrobial therapies. In this review, we provide an overview of the advantages of Ns-AMPs in improving therapeutic efficacy and biological stability, reducing side effects, and gaining the effect of organic targeting and drug controlled release. Then the different material categories of Ns-AMPs are described, including inorganic material nanosystems containing AMPs, organic material nanosystems containing AMPs, and self-assembled AMPs. Additionally, this review focuses on the Ns-AMPs for the effect of biological activities, with emphasis on antimicrobial activity, biosecurity, and biological stability. The “state-of-the-art” antimicrobial modes of Ns-AMPs, including controlled release of AMPs under a specific environment or intrinsic antimicrobial properties of Ns-AMPs, are also explicated. Finally, the perspectives and conclusions of the current research in this field are also summarized.

## Introduction

Antibiotics were considered as one of the most important weapons in the treatment of bacterial, fungal, and some other infectious diseases. During the 1940s, they have saved millions of lives and have prolonged the life span for a couple of years (Durand et al., 2019; Gajdács, 2019). However, the injudicious use of antibiotics in clinical treatment and other fields is threatening worldwide safety aspects on health seriously because of the growing drug resistance. When microbes are treated with antimicrobial agents, it has been amply reported that the development of bacterial resistance can be performed in a number of ways. In addition to producing β-lactamases, altering drug binding sites, or penetrating cell membrane, microbes can also utilize the quorum sensing (QS) to induce the formation of biofilm, preventing the interaction with antimicrobials (Kalia et al., 2019; Parasuraman et al., 2020). At the same time, irrational and inappropriate use of antibiotic in many fields such as healthcare, agriculture, and biomedicine contributed to advantages for resistant microbes to emerge, spread, and persist (Holmes et al., 2016; Ma et al., 2020). Colistin is considered to be the last line of defense against pathogen infections caused by the carbapenem-resistant *Enterobacteriaceae* due to the prevailing wisdom of low probability of chromosomal-mediated drug resistance in the bacterial family (Ma et al., 2020). MCR-1 is a new gene found in nature that makes bacteria resistant to polymyxin, and it is responsible for plasmid-mediated colistin resistance. Furthermore, many reports have identified that this transmissible resistance mechanism is rapidly distributed (McGann et al., 2016; Stoesser et al., 2016). Hence, the golden age for antibiotics to treat pathogenic infection is over, and the lookout for new alternatives to antibiotics is pressing.

**FIGURE 1:**
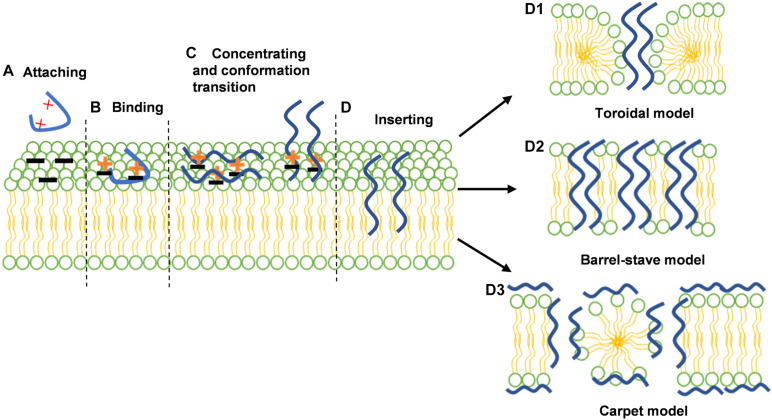
The interactions between antimicrobial peptides (AMPs) and bacterial membranes; **(A)** AMPs are attracted with the negatively charged surfaces of Gram-negative (outer membrane) or Gram-positive (cell wall) bacteria by electrostatic attraction; **(B)** AMPs exerts a binding process with the lipopolysaccharide (LPS) of Gram-negative or lipoteichoic acid (LTA) of Gram-positive bacteria; **(C)** AMPs start to accumulate on the bacterial membrane surface and adopt their stable secondary structure; **(D)** AMPs insert into lipid bilayer and exert various membrane disruptive models of AMPs: toroidal models **(D1)**, barrel-stave model **(D2)** carpet model **(D3)**. the membrane is demonstrated by two colors: green represents the phospholipid head and yellow represents the tail. The AMPs are shown in the curve, highlighted in blue. Reproduced with permission (Wang et al., 2019b). Copyright 2018, Wiley.

For decades, considerable efforts have been devoted to the development of novel antimicrobials to replace the crucial role of antibiotics. Antimicrobial peptides (AMPs), also termed host defense peptides (HDPs), are small proteins with 2 to 100 amino acids in length with antimicrobial, antiviral, and antineoplastic activity (Lazzaro et al., 2020; Wang et al., 2020). The sequence component simplicity of AMPs makes them light on evolving *de novo*. Some tertiary structures (e.g., defensins) have evolved continuously in species as diverse as microbes to plants, to vertebrates, and invertebrates, and remain appreciable against many pathogens (Broekaert et al., 1995; Gordon et al., 2005; Shafee et al., 2017). Furthermore, AMPs neutralize the bacterial endotoxins, impair the effect of inflammation and repair wound tissue by accelerating angiogenesis and re-epithelialization. More importantly, Spohn et al. found that resistance levels induced by AMP controls were significantly lower than the resistance levels evoked by antibiotic controls (Spohn et al., 2019). These advantages further indicated the potential of AMPs as substitution of antibiotics (Yang et al., 2019; He et al., 2020). Mechanistically, the stereotypical mechanism of AMP action includes the following three steps, largely preventing the occurrence of bacterial resistance: First, the cationic of AMPs exerts a binding process with the negatively charged surfaces of Gram-negative (outer membrane) or Gram-positive (cell wall) bacteria (Figures 1A,B). Then AMPs start to accumulate on the bacterial membrane surface and adopt their stable secondary structure (Figure 1C). Last, as the increase in peptide-to-lipid ratio on the bacterial membrane, the hydrophobic region of AMPs gradually interacts with phospholipid heads on the bacterial membrane. When a threshold concentration is reached, AMPs would disrupt the bacterial membrane through the formation of open pores, causing cell lysis. The specific membrane disruptive actions are shown in Figure 1D. (Bechinger and Gorr, 2017; Juthani et al., 2019; Zhang W. et al., 2020). However, new pieces of evidence about bacterial resistance to AMPs are about to overturn this model. It has been demonstrated that the Gram-negative bacteria can decrease the negative charge of the membrane surface by the incorporation of 4-aminoarabinose (Ar4N) or palmitoylation in lipid A, preventing the interaction with AMPs. It may suggest that the membrane disruptive mechanism is not the reason why AMPs restrain the evolution of bacterial resistance. The latest results found that except for the interaction with bacterial membrane, AMPs may act intracellularly, including inhibition of DNA/RNA or protein synthesis (Scocchi et al., 2016). Thus, the multisite antimicrobial action of AMPs contributes to rapid sterilization so that the acquisition of AMP resistance becomes tough (Bechinger and Gorr, 2017).

Unfortunately, like other peptide drugs, AMPs also have some obstacles that hindered their efficacy *in vivo*: (a) AMPs are natural substrates for gastrointestinal degradation, thus, limiting oral bioavailability, (b) they exhibit cytotoxicity at higher concentration and immunogenicity, and (c) bioavailability of peptide drugs are also limited due to rapid metabolism through kidneys and liver (Smart et al., 2014; Walkenhorst, 2016). For these reasons, very few clinical trials have involved peptide drugs.

Recent studies have focused on nanotechnology to remedy the drawbacks of traditional free AMPs. Nanoparticles (NPs) are ultra-small particles in the size range of 0.1 to 100 nm, and their size is almost the same as antibodies, nucleic acids, and proteins in biomolecules. Depending on the easily modified and flexible physicochemical properties, NPs have reflected targeted therapy and excellent antimicrobial properties (Zhang W. et al., 2014; Otari et al., 2017, 2019; Patel et al., 2019a). Due to the high surface-to-volume ratios of NPs, the contact surface between NPs and bacteria membranes was increased, which resulted in the destruction of the cell permeability and physiological functions of membranes, then the disruption of cellular functions caused by interacting with intracellular proteins and DNA, generating reactive oxygen species (ROS) (Li et al., 2017; Torres et al., 2019; Zheng et al., 2019). Hence, the peculiar antimicrobial mechanisms of NPs are employed to kill pathogens quickly and further reduce, or are less prone to, bacteria resistance by multiple antibacterial models. In addition to enhancing the inherent antimicrobial properties of antimicrobials, controlled self-assembled nanostructures can be used as drug carriers to improve the retention and permeability effect (EPR) and achieve the effect of direct administration toward the target organ (Azzopardi et al., 2013). At infection spots, certain substances (such as protease and endotoxin) released by the bacteria trigger inflammatory mediators and activate immune responses that impair barrier function and widen gaps, leading to the concentration of NPs (Abdulkhaleq et al., 2018; Fenaroli et al., 2018). Therefore, NPs have become remarkable tools for targeted imaging, diagnosis, medical therapy, while imparting applications in healthcare, food safety, and agricultural production sciences (Dong et al., 2016). From an applied point of view, nanotechnology can be used to make AMPs form nanoparticles to overcome the disadvantages of AMPs, such as decreased renal clearance and proteolysis, extended half-life, and increased target selectivity (Huh and Kwon, 2011; Martin-Serrano et al., 2019; Walvekar et al., 2019).

**FIGURE 2:**
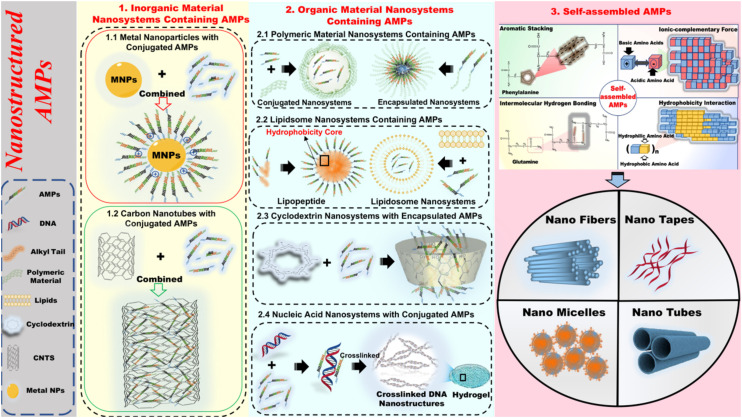
The specific classification and description of nanostructured antimicrobial peptide (Ns-AMPs). The Ns-AMPs could be classified into inorganic material nanosystems containing AMPs, organic material nanosystems containing AMPs, and self-assembled AMPs. Among them, the inorganic material nanosystems containing AMPs also can be classified into metal nanoparticles with conjugated AMPs and carbon nanotubes with conjugated AMPs. The organic material nanosystems containing AMPs can be divided into polymeric material nanosystems containing AMPs, liposome nanosystems containing AMPs, cyclodextrin nanosystems with encapsulated AMPs, and nucleic acid nanosystems with conjugated AMPs. The self-assembled AMPs could be classified into aromatic stacking, ionic-complementary force, intermolecular hydrogen bonding, hydrophobicity interaction.

Hence, we provide here an overview of different nanosystems formed on the basis of AMPs. Based on a variety of nanosystem modifications, nanostructured antimicrobial peptides (Ns-AMPs) could be categorized into three parts: inorganic material nanosystems containing AMPs, organic material nanosystems containing AMPs, and self-assembled AMPs. The specific classification and description are shown in Figure 2.

## Inorganic Material Nanosystems Containing Antimicrobial Peptides

### Metal Nanoparticles With Conjugated Antimicrobial Peptides

Metal nanoparticles (MNPs) have emerged as promising antimicrobial vectors against ESKAPE pathogens in the last decades due to their potent therapeutic potential, readily adjustable shape and size, manageable drug delivery and release, and less probability of microbial resistance (Gupta et al., 2021). The studies about MNPs with conjugated AMPs are versatile and can be categorized into gold nanoparticles (Zheng et al., 2019), silver nanoparticles (Gao et al., 2020), alumina nanoparticles (Torres et al., 2019), ruthenium nanoparticles (Huang et al., 2017), copper nanoparticles (Ishida et al., 2020), hybrid nanoparticles, etc., depending on the types and state of metal nanoparticles (Yang et al., 2020). Among all MNPs, silver (Ag) and gold (Au) nanoparticles with conjugated AMPs have been widely studied and used due to their appealing antimicrobial activity.

Silver nanoparticles (AgNPs) have been considerably applied in the antimicrobial field based on the strong absorption function against microbes (Hamida et al., 2020). However, because of the instability of AgNPs in an aqueous solution, the silver ions released by AgNPs also cause cytotoxicity of eukaryotes dramatically (Cho et al., 2013). Thus, most studies have modified the AgNPs via conjugated peptides to improve the biosafety *in vivo*.

**FIGURE 3:**
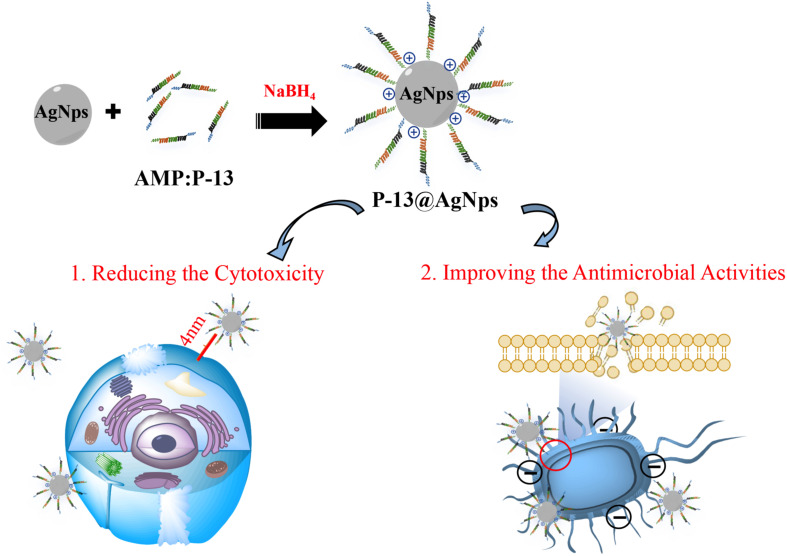
Mechanisms for antimicrobial properties of P13@silver nanoparticles (AgNPs) (cytotoxicity and antimicrobial activity). The peptide P13 increases the distance between AgNPs and cells, which significantly reduces the cytotoxicity. Meanwhile, the peptide P-13 improves the cationic on the surface of AgNPs, promoting the interaction of P-13@AgNPs with bacteria membrane, causing the death of microbes.

Gao et al. (2020) recently described that the AgNPs protected by the P-13 (KRWWKWWRRCECG) can efficiently deal with pathogens (*Escherichia coli*, *Staphylococcus aureus*, and *Bacillus pumilus*) with the minimum inhibitory concentrations (MICs) up to 7.8 μg/ml. The P-13@AgNPs exhibited excellent antimicrobial activity, and the MIC values of P-13 and AgNPs against tested pathogens were about 2–64 times higher than that of P-13@AgNPs. Meanwhile, the P-13@AgNPs significantly reduced the NIH-3T3 cell cytotoxicity caused by intrinsic AgNPs, reflecting the better potential for clinical treatment (Figure 3). With a similar approach, Falanga et al. (2020) studied the cytotoxicity of the pristine AgNPs and indolicidin-decorated AgNPs, suggesting that AgNPs evoke cytotoxicity seriously depending on their surface chemistry. Still, the AgNPs decorated by peptide-indolicidin (ILPWKWPWWPWRR) can improve the surface properties of AgNPs, significantly reducing the cytotoxicity. Additionally, except for stable conjugation between AgNPs and AMPs, D’Souza et al. (2020) executed the Ag ion-controlled release system by inserting an unnatural amino acid, 3′-pyridyl alanine (3′-PyA), into the peptide sequence. The addition of the Ag ions enables peptides to form a stable hydrogel nanostructure, inducing the encapsulation of Ag(I) in an aqueous solution. The results showed that the L9(3′-PyA)-Ag hydrogels [(3′-PyA)LRLRLRL(3′-PyA)] could efficiently kill *E. coli* and *S. aureus*, and its antimicrobial properties are roughly the same as Ag(I). As the minimal release of Ag ions is less than 4%, the L9(3′-PyA)-Ag hydrogels hardly cause toxicity and hemolysis on fibroblasts and red blood cell.

Gold nanoparticles (AuNPs) are one of the most representative inorganic materials. They have attracted more attention in immunoassay application, fluorescence imaging, specific delivery of siRNA, and targeting cancer cells *in vivo* due to their versatile structural properties and optical characteristics (Peng et al., 2016; Pranantyo et al., 2019; Liu et al., 2020). Meanwhile, in contrast to AgNPs with high cytotoxicity, the high stability and chemical inertness of the AuNPs exhibit an excellent biocompatibility, and it has been documented that the AuNPs with a size below 2 nm displayed the antimicrobial activity via bacterial membrane destruction, DNA damage, and the production of reactive oxygen species (ROS) (Zheng et al., 2018). Thus, it has inspired to further explore the functionalized bactericidal properties of AuNPs coated with antimicrobials (Jin et al., 2016; He et al., 2019; Makowski et al., 2019).

**FIGURE 4:**
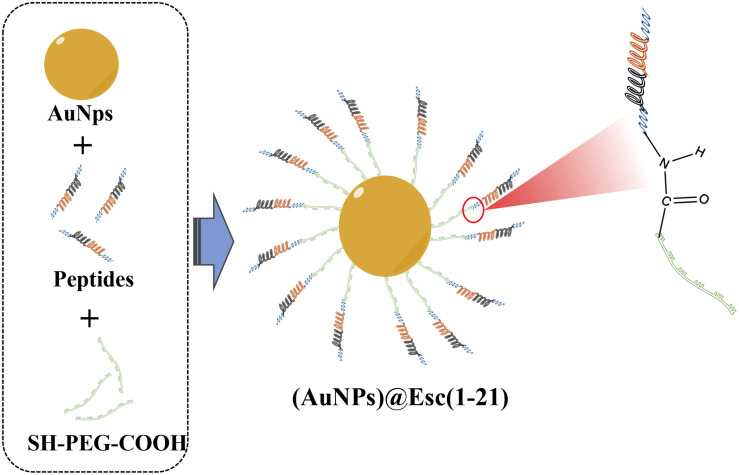
The composition of gold nanoparticles (AuNPs)@Esc(1-21).

Otari et al. designed an Au–peptide–alginate biohydrogel containing AuNPs, alginate cross-linked polymer, and thermostable nisin that could form spherical nanostructure with peptides-nisin presenting on their surfaces. The biohydrogel exhibited effective antimicrobial activity against pathogens (*S. aureus*, *B. cereus*, and *E. faecalis*) and synergistic antimicrobial effect with AuNPs. Furthermore, the biohydrogel displayed catalytic activity in reducing 4-nitrophenol and hexacyanoferrate (III), demonstrating that it is an ideal example of rapid, green, and cost-effective approaches for synthesizing metal nanoparticles (Otari et al., 2016). In addition, Casciaro et al. (2017) described an AuNP–AMP conjugate as shown in Figure 4. Esc(1-21) (GIFSKLAGKKIKNLLISGLKG-NH_2_), a derivative of the frog skin AMP esculentin-1a, is attached to AuNPs through poly(ethylene glycol) to overcome high toxicity and low stability. The results showed that the AuNPs@Esc(1-21) hardly generated toxicity toward human keratinocytes and exhibited better resistance to proteolytic digestion. In particular, compared with those of free peptide, the antibacterial activity of AuNPs@Esc(1-21) against *P. aeruginosa* increased 15-fold. Additionally, the conjugation of AuNPs with AMPs has proven to be an efficient strategy to protect against bacterial infections. Hybrid nanostructures (DAP–AuNP) formed by bonding between AuNPs and daptomycin (Dap, a cyclic lipopeptide AMPs) maintained inherent sterilization properties and triggered excellent synergistic effect toward methicillin-resistant *S. aureus* (MRSA) simultaneously (Zheng et al., 2019). The results of the antibacterial mechanism confirmed that localized Dap in the internal nanostructure would form more and larger holes on the bacterial membrane, leading to the rapid entry of Dap–AuNP into bacterial cells. Then, Dap–AuNC further induces ROS accumulation by motivating the bacterial DNA damage, ultimately leading to the death of the pathogens.

Taken together, the cytotoxicity development of AgNPs is a huge impediment for clinical applications. It is a positive strategy that the conjugate of AMPs with AgNPs efficiently impaired the cell toxicity of mammals through the surface modification of AgNPs, and the delayed release of Ag(I) continued the inherent excellent antimicrobial properties, simultaneously. Furthermore, some literature (Yang et al., 2020) reported that AMPs combined with AgNPs and AuNPs to form AMP–Au/AgNP hybrid nanocages, further weakening cytotoxicity. Interestingly, most recent studies demonstrated that MNPs and AMPs have a strong synergistic effect against a variety of pathogens (Albada and Metzler-Nolte, 2017; Huang et al., 2017; Pal et al., 2019). Therefore, the appropriate combination of AMPs with MNPs to treat multidrug-resistant bacteria and increase the abundance of antibacterial drugs will be the future development trend.

### Carbon Nanotubes With Conjugated Antimicrobial Peptides

Carbon nanotubes (CNTs), formed by coaxial rolled-up tubular lamellae of graphene, have been proven to be the most promising carbon-based materials in medicine and electronic aspects, based on the unique structure and physicochemical properties. Generally, the lamellae of CNTs have a different number of plies, which can be categorized into single-walled (SWCNT) and multi-walled (MWCNT) (Iijima, 1991; Bianco et al., 2005). With the emergence of bacterial resistance induced by antibiotic, the combination of CNTs with AMP agents has attracted lots of attention in biopharmaceutical fields. It has been documented that the surface of CNTs can decorate various functional groups and provides binding sites, thus, improving the dispersibility and biocompatibility of uploaded drugs (Zhang et al., 2012). Pradhan et al. (2017) reported that SM-CNTs conjugated to LL-37 (LLGDFFRKSKEKIGKEFKRIVQRIKDFLRNLVPRTES) or indolicidin by the use of 1-(3-dimethylaminopropyl)-3-ethylcarbodiimide and N-hydroxysuccinimide (EDC-NHS) conjugation protocol to impair side effects. The results demonstrated that the two-peptide conjugation with CNT was 1,000-fold lower than the dosage of free AMPs required for intracellular biological functions, suggesting that SM-CNTs improved the immune-modulatory efficacy of LL-37 and indolicidin, while efficiently reducing the dosage of peptide agents. Additionally, CNTs had been employed to absorb and load different types of the drug due to their large specific surface area and length (Liu et al., 2007). Xu M. et al. (2020) designed multifunctional conductive macroporous nanocomposite hydrogels (MNHs) formed by CNTs and gelatin methacryloyl through air-in-water emulsion template to retain the regeneration of soft tissue and load AMP-HHC36 (KRWWKWWRR) with bactericidal capacity. The cell assay *in vitro* revealed that the NE-4C neural stem cells still conducted spreading and differentiation in MNHs. Meanwhile, the MNHs released the AMPs continuously, and its release concentration can efficiently kill *S. aureus*, exhibiting significantly antimicrobial properties and wound healing efficiency *in vivo*.

Based on their characteristics, CNTs have many advantages, but the biosafety problem *in vivo* also needs to be considered due to its designation as a 2B type carcinogen. The knowledge systems about the decorated methods of surface and self-performances of CNTs are still deficient. Table 1 summarizes the information related to inorganic material nanosystems containing AMPs described in this section.

**TABLE 1:** Most representative examples of inorganic material nanosystems containing antimicrobial peptides (AMPs).

AMP	Sequence	Combined materials	Antimicrobial activity	Tested microorganisms	Special functions	References
P-13	KRWWKWW RRCECG	AgNPs	MIC: 7.8–15.6 μg/ml	*E. coli, S. aureus, P. aeruginosa, B. pumilus*	Reduces the cytotoxicity on mouse fibroblast NIH-3T3 cells, has broad-spectrum antimicrobial activity	Gao et al., 2020
Indolicidin	ILPWKW PWWPWRR	AgNPs	−	−	AgNPs coated with indolicidin delays the Ag + release and reduces the toxicity for *D. magna*, *R. subcapitata*, and plant seeds	Falanga et al., 2020
L9	(3′-PyA)LRLRLRL(3′-PyA)	AgNPs	Eliminates the bacterial cells (OD_600_ < 0.1)	*E. coli* and *S. aureus*	Has broad-spectrum antimicrobial activity, no cytotoxicity fibroblast NIH-3T3 cells, has antimicrobial synergistic effect between L9 and AgNPs	D’Souza et al., 2020
Daptomycin	−	AuNPs	Daptomycin with a concentration of 10 μg/ml eliminates all of the bacterial cells after an incubation of 1 h	MRSA	Has antimicrobial synergistic effect between daptomycin and AuNPs	Zheng et al., 2019
Esc(1-21)	GIFSKLAGKKIKN LLISGLKG-NH _2_	AuNPs and poly(ethylene glycol)	MBC: 0.08 μM/ml	*P. aeruginosa*	No cytotoxicity to human keratinocytes, has resistance to proteolytic digestion, has the ability for eradication of biofilm	Casciaro et al., 2017
PEP	CACWQVSRRRRG	AuNPs	AuP@TAT/PEP with content of peptide about 2.5% eliminates the bacterial cells after 25 h incubation (OD_600_ < 0.06)	*E. coli* and *S. aureus*	Can be used as a carrier for *in vivo* gene, activation in tissue regeneration, promotes the gene transfection efficiency in rat mesenchymal stem cells	Peng et al., 2016
CopA3	LLCIALRKK-NH_2_	AuNPs and ginsenoside CK	−	−	Ameliorates LPS-induced nitric oxide and reactive oxygen species production and suppresses the mRNA and protein expression of pro-inflammatory cytokines in macrophages. inhibited the activation of the nuclear factor-κB (NF-κB) and mitogen-activating protein kinase (MAPK) signaling pathways NF-κB	Liu et al., 2020
LL-37	LLGDFFRKSKEKI GKEFKRIVQRIKDFL RNLVPRTES	CNTs	MIC: 0.02 μg/ml	*Salmonella typhimurium*	Enhances antimicrobial activity	Pradhan et al., 2017
HHC36	KRWWKWWRR	CNTs and gelatin methacryloyl	−	*S. aureus*	Promotes the spreading and differentiation of NE-4C neural stem cells	Xu M. et al., 2020

## Organic Material Nanosystems Containing Antimicrobial Peptides

It has been documented that nanotechnology is utilized to carry or modify AMPs, which can significantly enhance the therapeutic effect of peptides (Faya et al., 2018). The organic materials coating or covalently connecting with AMPs through a flexible design turned out to provide firm, porous, and regular shielding structures, which had a number of advantages, including the improvement of antibacterial activity, protease stability, biological safety, targeted ability, and controllable pharmacokinetic characteristics (Nordström and Malmsten, 2017). Nowadays, there are many polymers such as natural or synthetic polymers, biodegradable polymers, liposomes, cyclodextrins, DNA, etc. These abundant organic materials provide reliable support for different applications of AMPs (Martin-Serrano et al., 2019; Patel et al., 2019b; Kalia et al., 2021). Here, a summary of the current situation of the organic material nanosystems containing AMPs was given, with particular emphasis on related biological effects of AMP-containing formulations and the interactions of the inner systems. Table 2 summarizes the information related to organic material nanosystems containing AMPs described in this section.

**TABLE 2:** Most representative examples of organic material nanosystems containing AMPs.

AMP	Sequence of AMPs	Combined ways	Combined materials	Antimicrobial activity	Tested microorganisms	Special functions	References
Aurein 2.2	GLFDIVKK VVGALC	Conjugation	Hyperbranched polyglycerol	MIC: 110 and 120 μg/ml.	*S. aureus* and *S. epidermidis*	−	Kumar et al., 2015
K4	KKKKPLF GLFFGLF	Conjugation	PLGA	K4 with concentration of 100 μg/ml inhibits the bacterial growth by 40% and 30%, respectively.	*S. aureus* and *P. aeruginosa*	Has the function of drug sustained release	Vijayan et al., 2019
HHC10	H-KRWWK WIRW-NH2	Conjugation	PEG	−	*S. aureus*, *S. epidermidis*, and *E. coli*.	Improves stability in human serum	Cleophas et al., 2014
KLAK	CGGGKLAK LAKKLAKLAK	Conjugation	Chitosen-PEG	−	*S. aureus*	Delays the AMP release, targeted gelatinase-positive bacterial species	Qi et al., 2017
OH30	KFFKKLKN SVKKRAK KFFKKPRV IGVSIPF	Encapsulation	Carboxymethyl chitosan	The OH30 and CMCS at proportion by weight 1:2, which kills the bacteria growth nearly 100% within 24 h	*E. coli*.	Has the function of drug sustained release, enhances cell migration and promotes wound healing, induces IL10 expression	Sun T. et al., 2018
17BIPHE2	−	Encapsulation	Pluronic F127/PCL Poly (εcaprolactone)	The bacteria colonies of four strains was reduced with 3.2, 3.6, 3.8, and 3.6-log, respectively, after incubation with 17BIPHE2 for 2 h.	*MRSA*, *K. pneumoniae*, *A. baumannii*, and *P. aeruginosa*	Has the function of drug sustained release, has no cytotoxicity to skin cells and monocytes, could eliminate the bacterial biofilms	Su et al., 2019
SET-M33	(KKIRV RLSA)_4_K_2_ KβA-OH	Encapsulation	Dextran	MIC: 16 μg/ml	*P. aeruginosa*	Improves the residence time and keeps the concentration of AMPs in the lungs, reduces toxicity toward macrophages and epithelial cells	Falciani et al., 2020
Nisin	ITSISLCT PGCKTGA LMGCNMKTA TCHCSIHVSK	Encapsulation	[Poly (l-lactide)-graft-chond roitin sulfate (PLLA-g-CS) copolymers]	Average inhibition zones (mm) = 16–17.6	*S. aureus* and *E. coli*	Has the function of drug sustained release. No cytotoxicity to human dermis fibroblast cells	Ghaeini-Hesaroeiye et al., 2020
S32	PAMAM-(LV)_32_	Conjugation	Poly(amido amine) dendrimers (PAMAM)	MIC: 0.04–0.21 μM/ml	*E. coli*, *P. aeruginosa*, and *Klebsiella pneumoniae*	Improves the stability in serum and divalent cations at physiological concentrations	Lam et al., 2016
DJK-5	^*D*^(VQWRA IRVRVIR)	Encapsulation	Hyaluronic acid	−	*P. aeruginosa*	Reduces the cytotoxicity of epithelial tissue	Kłodzińska et al., 2019
RBRBR	RBRBR (B is l-4-phenyl-phenylalanine)	Encapsulation	Chitosan	Log CFU/ml of *S. aureus* treated with 5 mg/ml was 2.72–4.7	*S. aureus*, *MRSA*	Has the capacity of controlled drug release, reduces toxicity of human red blood cells, and eliminates biofilm	Almaaytah et al., 2017
Peptide + 2/peptide + 5	GLKEI FKAGLGSL VKGIAAHVAS/GLKRIFKS GLGKLVK GISAHVAS	Encapsulation	Liposomes coated with Eudragit E-100	MIC_*peptide+*__2_: 1.25 μM/ml MIC_*peptide+*__5_: 5 μM/ml	*E. coli* and *L. monocytogenes*	Enhances the antimicrobial activities of peptide + 2 and peptide + 5.	Cantor et al., 2019
DPK-060	GKHKNKGKKN GKHNGWKWWW	Encapsulation	LNCs, ML-LNCs, and cubosomes in poloxamer gel	MMC (minimum microbicidal concentration): 1.2–2.4 μg/ml	*S. aureus*	−	Håkansson et al., 2019
AP114	GFGCNGP WNEDDLR CHNHCKSIK GYKGGYCAKG GFVCKCY	Encapsulation	Cubic glycerol monooleate/water and hexagonal glycerol monooleate/oleic acid/water	MIC: 4–8 μg/ml	*S. aureus* and *MRSA*	Has the capacity of controlled drug release	Boge et al., 2016
MccJ25	GGAGHVPE YFVGIGTPISFYG	Encapsulation	Double-coated (pectin/WPI) liposomes	−	−	Has the capacity of controlled drug release, improves its stability in gastrointestinal digestion	Gomaa et al., 2017
HD5-myr	ATCYCRTGRCA TRESLSGVCE ISGRLYRLCCR-myr	Conjugation	N-terminal with alkyl tail	MBC: 6.25–12.5 μg/ml	*MRSA*, *E. coli*, *A. baumannii*, *P. aeruginosa*, and *K. pneumoniae*	Has broad-spectrum antimicrobial activity, reduces cytotoxicity	Lei et al., 2018
WMR2PA	WGIRRILK YGKRSAAAA AAK(C19)	Conjugation	C-terminal with alkyl tail	WMR2PA with concentration of 50 μM/ml has 40% and 70% inhibition rates against *P. aeruginosa*, *C. albicans*, respectively	*P. aeruginosa*, *C. albicans*	Eliminates bacterial biofilm, improves protease stability	Lombardi et al., 2019b
K9	(C16)WILAAGGG KKKKKKKKK-TAT	Conjugation	N-terminal with alkyl tail	MIC: 27–40 μM/ml	*E. coli*, *S. aureus*, *B. subtilis*, *P. aeruginosa*, and *MRSA*	Has broad-spectrum antimicrobial activity, penetrates the blood–brain-barrier to inhibit bacterial infection	He et al., 2018
	(C16)YEALRVA NEVTLN	Conjugation	Alkyl tail	−	*−*	Has the function of drug sustained release	Castelletto et al., 2017
LyeTxI	IWLTALKFLGK NLGKHLA LKQQLAKL	Encapsulation	β-CD	MIC: 7.81–15.62 μg/ml	Periodontopathic bacteria	Eliminates bacterial biofilm and prevents bacterial biofilm development	Cruz Olivo et al., 2017
Alamethicin	−	Encapsulation	γ-CD	MIC: 4.1563 mg/ml	*L. monocytogenes*	Increases alamethicin solubility and biocompatibility, has been applied for controlled release delivery of AMPs	Zhang et al., 2018
AMPs L12	LKKLLK KLLKKL	Conjunction	Polyanionic DNA	MBC: 8 μM/ml	*E. coli*, *S. aureus*, and *MRSA*	Has been applied for controlled release delivery of AMPs, has anti-inflammatory action, has cell selectivity for human dermal fibroblasts and HaCaT cells	Obuobi et al., 2019

### Polymeric Material Nanosystems Containing Antimicrobial Peptides

High polymer is a kind of macromolecule formed by repeated linking of specific structural units, with outstanding mechanical properties and stability, such as naturally occurring chitosan, hyaluronic acid, dextran, and polyethene glycol, poly(lactide-co-glycolide) (PLGA) synthesized by modern chemical techniques (Sun H. et al., 2018). Polymeric material nanosystems containing AMPs have many advantages of remarkable biocompatibility and biodegradability, controlled drug release, and lower toxicity of degradation products (Joseph et al., 2017) by which AMPs can acquire a broad development prospect in modern medicine, including wound dressings, implant coatings, tissue engineering, especially providing new hope for drug-resistant bacteria treatment, by combining into functional polymers to form nanoparticles, nanofibers, multilayers, even hydrogels, vesicles, and micelles. Here, we analyzed the latest typical cases and summarized the remaining challenges and unsolved problems in polymeric material nanosystems containing AMPs.

Due to the uncontrollable interaction between AMPs and polymers, most polymeric material nanosystems containing AMPs are compelled to manifest reduced antimicrobial efficiency. Kumar et al. (2015) found that after conjugating to hyperbranched polyglycerol with different peptides, MICs of Aurein 2.2 (GLFDIVKKVVGALC) against *S. aureus* and *S. epidermidis* both decreased from 16 and 32 μg/ml to 110 and 120 μg/ml, respectively. Polymer attached to the N-terminal of AMPs is more likely to form nanostructures, but antimicrobial activity is also affected by modifications to the active sites of AMPs (Wei et al., 2017). However, impressive studies have demonstrated that AMPs could maintain their antimicrobial activity in the nanosystems. Jiang et al. (2018) expanded traditional nanofiber membranes from 2D to 3D, loading and releasing LL37, to retain its original activity. In addition to encapsulation, conjunction could also cause the same result. LL-37-derived peptides (GFKRIVQRIKDFLRNLV) decreased the antimicrobial activity with methoxy PEG analog within 24 h, but increased cell selectivity and serum protease activity (Gong et al., 2017). Some materials, such as PLGA, can fully release a variety of active substances with simpler designs (Vijayan et al., 2019). Although the antimicrobial activity of AMP-K4 (KKKKPLFGLFFGLF) encapsulated in PLGA nanosystem was slightly weakened, the growth factor in this nanosystem efficiently promoted angiogenesis. High activity of AMP-HHC10 (H-KRWWKWIRW-NH_2_) was covalently combined with PEG hydrogel to form a mimetic with stable antibacterial activity after treatment with protease for 24 h (Cleophas et al., 2014).

Generally, polymeric material nanosystems containing AMPs slightly decreased the antibacterial activity of AMPs, but it is not worth paying excessive attention to this regard because the packaging characteristic and large size of the polymeric material nanosystems containing AMPs efficiently protect AMPs from capturing and clearing by the reticuloendothelial system and interference of salt ions or protease, improving their effective half-life and stability *in vivo*. Moreover, the nanosystem with the ability to control drug release promotes the drug concentration at the desired infection site, thereby indirectly improving antibacterial effects of the AMPs *in vivo* (Qi et al., 2017). The controlled drug release is deemed as the most common entry point to deal with prominent biofilm colonization on medical materials and to treat chronic wound infection. According to the therapeutic target of sustained-release nanosystems, they can be divided into three categories to date.

**FIGURE 5:**
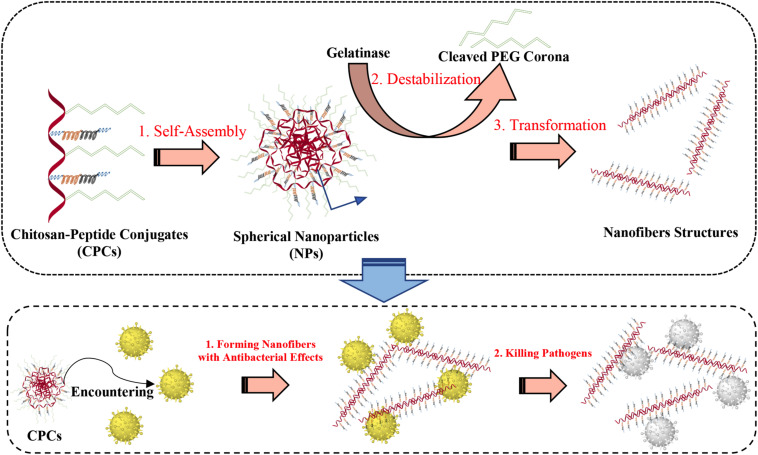
Illustration of the polymer–peptide conjugates and the principle of gelatinase-induced morphology transformation.

One is targeting and controlled release to kill infectious pathogens *in vivo*. The sensitivity to microbes is ensured by adding the enzyme digestion site in the nanosystems. For example, the polymer–peptide conjugates composed by chitosan backbone, AMP–KLAK (CGGGKLAKLAKKLAKLAK) and poly(ethylene glycol)-tethered enzyme-cleavable peptide could form spherical nanoparticles. Upon encountering bacteria that secretes the gelatinase, the poly(ethylene glycol)-tethered peptide is stripped from the nanosystem so that the polymer–peptide conjugates spontaneously transform from spherical nanoparticles to fibrous. The exposed AMP–KLAK exhibits antimicrobial activity, ultimately target killing the pathogens (Qi et al., 2017). The specific illustration is shown in Figure 5.

The second category targets pathological organs and tissues in the body, such as skin wounds, lungs, and intestinal tract, through novel administration, an emerging category of research known as targeted release *in vivo*. Sun T. et al. (2018) encapsulated the king cobra AMP–OH30 (KFFKKLKNSVKKRAKKFFKKPRVIGVSIPF) into carboxymethyl chitosan nanoparticles and controlled release for at least 24 h, which significantly facilitated wound healing. Pluronic F127/17BIPHE2-PCL core-shell nanofibers induced sustained release of human cathelicidin peptide 17BIPHE2 for 4 weeks, with better antimicrobial activity over MRAS (Su et al., 2019). Skin wounds are easy to administer, but the lungs of patients with pneumonia often face two issues: short duration of action and low concentration of drugs in the lesion. The aerosol system formed by AMPs-SET-M33[(KKIRVRLSA)_4_K_2_KβA-OH] and single-chain dextran nanoparticles markedly prolonged the residence time and increased the concentrations of drugs in the lungs (Falciani et al., 2020). Since the lungs contact with the air directly, AMPs can be delivered through aerosols better. Therefore, when polymer nanosystems are considered as drug-delivery systems, the concept of design should be related to the characteristics of organs. Another common target organ is the intestinal tract apart from lung and skin wounds. When the drug enters the intestine, the concentration of drugs decreases because of the acidic environment of the stomach, which is also a serious issue. Smart morphological changes caused by pH or temperature allow for specific delivery (Feng et al., 2021). A dual-responsive nanocarrier developed from [poly (l-lactide)-graft-chondroitin sulfate (PLLA-g-CS)] copolymer nanogels could release 25% to 98% nisin according to the changes of pH and temperature in the mimic environment of the gastrointestinal tract (Ghaeini-Hesaroeiye et al., 2020).

Last, polymer nanosystems can target organs as well as target non-organ implants *in vivo*, such as bone grafts, implants (Chen et al., 2016), and catheters (Raman et al., 2014). With the emergence of drug-resistant bacteria, biofilms attached to implants *in vivo* become increasingly difficult to remove, increasing the risk of infection. Polymer nanosystems can adapt to the complex environment in the body, forming multilayers that attach to the implant and continuously release AMPs to eliminate biofilms, thereby alleviating infection.

**FIGURE 6:**
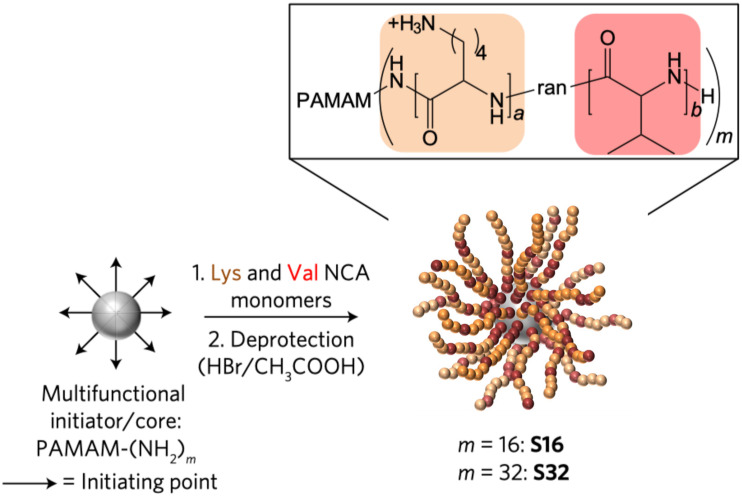
Synthesis of SNAPPs (Lam et al., 2016). Copyright 2016, Macmillan Publishers Limited, part of Springer Nature.

Additionally, polymeric nanosystems can improve the stability of AMPs. Lam et al. (2016) designed a star-shaped peptide (Figure 6) polymer nanoparticles polymerized with lysine and valine as polymers that exhibited good antimicrobial activity against serum and physiological salt concentrations (Lam et al., 2016). High cytotoxicity is the primary reason that hinders the application of AMPs, and polymer nanosystems can limit the dissociation of AMPs, hide the hydrophobic regions, weakening the binding capacity with the eukaryote membrane. The toxicity of the anti-biofilm AMP-DJK-5 [^*D*^(VQWRAIRVRVIR)] encapsulated by hyaluronic acid nanogels was four times lower than that of monomer peptides (Kłodzińska et al., 2019). However, none of the options is set in stone, and some designs can increase antimicrobial properties and obtain controlled drug release capabilities simultaneously. Almaaytah et al. (2017) designed a chitosan-based nanoparticle with AMP-RBRBR that can release AMPs for 14 days while reducing the toxicity of mammalian cells at the same time. Compared with the negative control, the antibacterial kinetics results was reduced by 5-log, and biofilm formation was inhibited about 98% (Almaaytah et al., 2017).

Different types of macromolecular polymers and their respective characteristics (such as pH sensitivity of sodium alginate) make them the most thoroughly studied, flexible, and functional among all Ns-AMPs. AMPs can be covalently linked to the polymer as a whole or coated by the polymer, with the principle to improve the antimicrobial activity, stability, and biological safety by forming a stable structure in the physical and chemical environment, increasing the local concentration of AMPs and protecting the hydrophobic region. In addition, the selection of different polymer materials to achieve controlled release of drugs addresses the issue of sustained antimicrobial activity against drug-resistant bacteria and biofilms, as well as targeting organs *in vivo*. At present, the interaction of AMPs, polymers, and the environment is a difficult point. Personally, the development of intelligent and transformable polymer nanosystems for *in vivo* application will be the focus of this research.

### Lipids Nanosystems Containing Antimicrobial Peptides

Lipid nanosystems include liposomes and lipopeptides. Liposomes are nanoscale hollow spherical artificial vesicles composed of one or multiple concentric lipid bilayers and range in size from 50 to 450 nm, encapsulating a hydrophilic compartment (Bozzuto and Molinari, 2015). Liposomes can be classified by the lamellarity, including unilamellar vesicles (ULVs), multilamellar vesicles (MLVs), and liquid crystalline nanoparticles (LCNPs). ULVs is very suitable for the encapsulation of hydrophilic drugs due to the large aqueous core, and MLVs tend to carry low soluble drugs (Immordino et al., 2006). Liposomes are similar to cell membranes and can carry a variety of substances, making them an almost ideal drug delivery system. Because liposome formulation has been commercialized or are in clinical trials, numerous applications of liposomes have been reported, such as antibiotics, anticancer, gene therapy (Chen C. et al., 2019; Narita et al., 2019), and some drugs like DepoCyt^®^, DaunoXome^®^, and Marqibo^®^ (anti-cancer) (Flühmann et al., 2019).

Antimicrobial peptides are encapsulated in the hydrophilic core of ULVs and MLVs, hindering the interaction of AMPs with bacterial membrane, displaying antimicrobial activity only when they are released from liposomes. Therefore, adjusting the controlled-release rate of AMPs is vital for exerting antimicrobial capacity (Hosta-Rigau et al., 2013), and the solutions to the above issues are described as follows: First, selecting reasonable hydrophobic AMPs is a way to regulate the controlled-release rate of AMPs due to the high permeability of liposomes to hydrophobic drugs. Cholesterol insertion then improved the rigidity of liposomes and enhanced the encapsulation effect of AMPs, further delaying the sustained release rate of AMPs. Similarly, liposome membranes combine with other molecules such as PEG and DNA, can achieve other functions, such as biosensing drug carriers.

**FIGURE 7:**
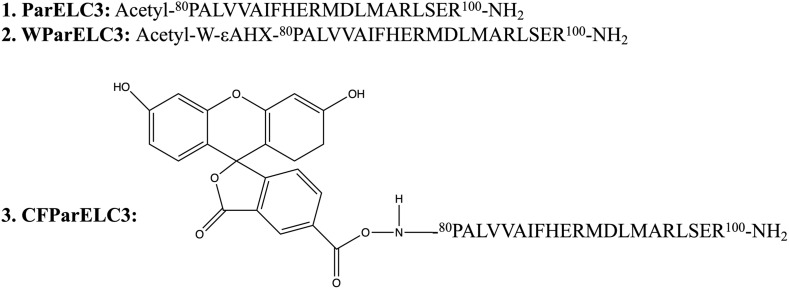
Primary structure of synthetized peptides (εAHX = ε-aminocaproic acid).

Interestingly, liposomes can improve the antimicrobial activity of AMPs in non-membrane disruption mechanisms. For example, peptide-ParELC3 encapsulated by rhamnolipid-based liposomes exhibits better antibacterial activity than the non-membrane disruption mechanism peptide-ParELC3 because rhamnolipid-based liposomes can be integrated into bacterial membrane smoothly. Peptide-ParELC3 derived from ParE toxin is carried through bacterial membrane to target topoisomerase, resulting in bacterial death. The primary structure of peptide-ParELC3 (acetyl-^80^PALVVAIFHERMDLMARLSER^100^-NH_2_) is shown in Figure 7 (Sanches et al., 2021). In addition, the combination of liposomes and cationic nanomaterials promotes the antibacterial properties of AMPs. It has been proven that compared with free peptide, the antibacterial activity of liposomes containing Peptide + 2 and Peptide + 5 can increase by approximately 2,000 times after being coated with a cationic polymer (Eudragit E-100) (Cantor et al., 2019).

As a special type of liposomes, liquid crystalline nanoparticles (LCNPs) consist of lipid bilayers, such as cubosomes and hexosomes, folded to acquire 2D and 3D structures with water channels interwoven with each other. In general, antimicrobial activity can decrease after AMP encapsulation. For example, the antimicrobial effect of cubosomes loaded with peptide AP114 (Boge et al., 2016) and DPK-060 (GKHKNKGKKNGKHNGWKWWW) (Boge et al., 2017; Håkansson et al., 2019) against *S. aureus* and *E. coli* was almost constant. Compared with LL-37 with broad-spectrum activity, cubosomes loaded with LL-37 displayed a narrow-spectrum bacterial effect, which were only active against Gram-negative strains (Boge et al., 2019).

Although the antimicrobial activity of AMPs decreases, the stability and selectivity of cells can be improved after liposome encapsulation. Liposome encapsulation can protect AMPs from enzymatic degradation and interference between salt ions and serum before reaching target tissues, such as tumor or infection site. Therefore, a high concentration of AMPs can be released in target tissues to achieve good therapeutic effects (Akbarzadeh et al., 2013). The degradation rate of double-coated (pectin/WPI) liposomes containing AMPs-MccJ25 (GGAGHVPEYFVGIGTPISFYG) in 2 h was lower than that of single coated or non-coated liposomes, which encapsulate the AMPs, reducing the contact of AMPs with healthy cells in the blood circulation, thereby, reducing the toxicity of AMPs (Bozzuto and Molinari, 2015; Gomaa et al., 2017). Peptide-ParELC3 derived from ParE toxin in rhamnolipid-based liposomes had no cytotoxicity to HepG2 cells, even at 1.3 and 50 μmol/L (Sanches et al., 2021). The toxic peptide melittin can cause allergic reaction and pain. Mao et al. found that melittin encapsulated in poloxamer 188 and nanoliposomes could reduce inflammation and allergy significantly, killing hepatocellular carcinoma (Mao et al., 2017).

**FIGURE 8:**
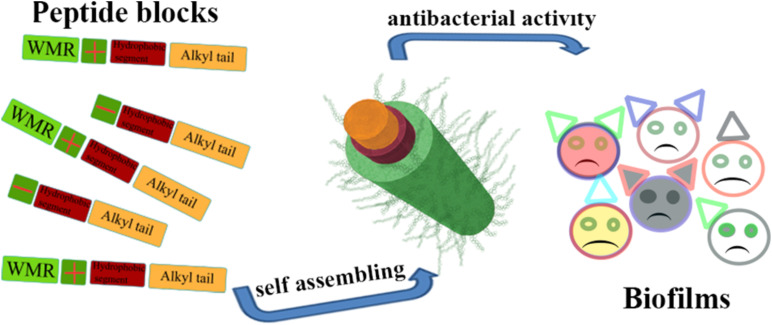
Illustration of the nanostructure of lipopeptide and its special function (Lombardi et al., 2019b). Copyright 2019, American Chemical Society.

Lipopeptides are formed by covalently binding AMPs to alkyl chains. Some studies have shown that lipopeptides significantly promote antimicrobial activity and eliminate bacterial biofilms due to enhanced hydrophobicity. Meanwhile, the alkyl chain of lipopeptides provides a hydrophobic core, triggering lipopeptides to self-assemble, thereby improving the stability of proteases and serums (He et al., 2018; Lei et al., 2018; Stachurski et al., 2019). However, it has been reported that the conjugated position of alkyl chain with peptide sequence is also crucial to the antimicrobial activity of AMPs. Alkyl chains attached to the C-terminus of antimicrobial peptides are always more effective than those to N-terminus of AMPs because alkyl chains block action sites of AMPs. In addition, lipopeptides also gain other additional capabilities, such as cell penetration or pH sensitivity by binding to functional fragments (Guo et al., 2010; Castelletto et al., 2017). Some typical examples are listed below. Compared with peptide HD5, C-terminally myristoylated HD5 (ATCYCRTGRCATRESLSGVCEISGRLYRLCCR-myr) assembled into nanobiotics had broad-spectrum bactericidal activity *in vitro*, which improved the destruction ability to *E. coli* and MRSA cell wall and membrane (Lei et al., 2018). For short peptides, Stachurski et al. studied that short-chain lipopeptides could form surfactant-like structure and preferentially targeted and penetrated microbial membranes causing potent antimicrobial activity (Stachurski et al., 2019). Lipopeptides can also improve the stability of proteases and eliminate biofilms. Lombardi et al. (2019a, b) described a stable nanofiber formed by combining the C-terminus of hagfish peptide WMR with lipopeptides significantly inhibited the formation of *P. aeruginosa* and *C. albicans* biofilm (Figure 8). The nanofiber remained stable in trypsin after incubation for 4 h, but WMR was rapidly hydrolyzed into two peptide fragments after another 1 h (Lombardi et al., 2019b).

After being attached to some functional segments, lipopeptides performed additional functions, such as penetration and pH sensitivity of cells. Bi et al. linked cell-penetrating peptide TAT and fatty acids to C- and N-terminal of non-alysine K9 [(C16)WILAAGGGKKKKKKKKK-TAT], respectively, with an exposed TAT shell, which could penetrate the blood–brain barrier and enter the bacteria to treat brain infection (He et al., 2018). Lipopeptides loaded with hydrophobic drugs can be released at specific pH values. Guo et al. constructed a fabric consisting of polyhistidine and polyarginine conjugated with cholesterol, which loaded the hydrophobic anticancer drug DOX. At a pH of 7.4, the fabric was inseparable, which could release DOX at pH 5. Additionally, the morphology of lipopeptides will be changed by pH value. It has been reported that the N terminus of the bioactive motif peptide block (YEALRVANEVTLN) conjugated to dodecyl, tetradecyl, or hexadecyl lipid chains could self-assemble into beta-sheet nano-tapes above the critical aggregation concentration. At a pH of 10, 1 wt% lipopeptide solution formed long, straight fibers. After the injection of 1 M HCl, peptide residues could become independent and stable hydrogels due to changes in the electrostatic charge (Castelletto et al., 2017).

In summary, both liposomes and lipopeptides contribute to the stability of AMPs. Liposomes can be considered as an ideal drug delivery system because of their remarkable biocompatibility and drug encapsulation ability, while lipopeptides have environmental sensitivity, which can significantly improve the antimicrobial activity of AMPs. Thus, lipopeptides are the most common covalent modification of AMPs. However, there are still issues to be solved in lipid nanosystems, including strong toxicity of lipopeptides and weak antimicrobial activity of liposomes. Furthermore, the controlled release of AMPs in liposomes and the molecular design of multifunctional lipopeptides also appear to be tremendous potential for being proceeded with.

### Cyclodextrin Nanosystems With Encapsulated Antimicrobial Peptides

Cyclodextrin is a cyclic oligosaccharide produced by amylose in the action of cyclodextrin glycosyltransferase, including α-CD, β-CD, and γ-CD, containing 6, 7, and 8 glucose units, respectively. CD is a slightly conical circular ring with hydrophilic outer edge and hydrophobic inner cavity, combining with organic and inorganic molecules, to form nanosystems. It can improve the stability, solubility, and dissolution rate of hydrophobic materials, reducing the side effects of peptides and achieving the controlled drug release effect, with an antibacterial and anti-inflammatory effect, such as the ingredients in aloe vera gum (Zhang et al., 2018; Gao et al., 2019; Lu et al., 2019; Xu Y. et al., 2020). Therefore, CD has been used in the field of cosmetic, medicine, and food, with great value in the field of AMP coating.

α-CD-based polypseudorotaxane hydrogels are novel functional materials with excellent biocompatibility, thixotropic nature, and reversible and stimuli-responsiveness properties (Domiński et al., 2019). Cruz Olivo et al. (2017) found that the associating compound LyeTxI/β-cyclodextrin (βCD) had a better eliminating effect on multispecies biofilms than that of free LyeTxI (IWLTALKFLGKNLGKHLALKQQLAKL). Zhang et al. (2018) encapsulated alamethicin (ALM) with γ-CD in a 1:1 molar ratio and that of MICs was 0.429 mg/ml.

Although CD derivatives have acquired satisfactory results as effective drug carrier materials at the laboratory level, their practical clinical application still faces many challenges, including biological toxicity, application range, and so on. Effective loading rate and green chemistry during synthesis are imminent issues (Tian et al., 2020).

### Nucleic Acid Aptamer Nanosystems With Conjugated Antimicrobial Peptides

Nucleic acid aptamers, known as chemical antibodies, are unique 3D structures composed of 20–80 nucleotides with specific targeting ability, high binding affinity, low immunogenicity, and easy modification. AMPs could be embedded into RNA or single-stranded DNA molecules to form nucleic acid aptamer-functionalized systems, which have been extensively applied in various fields (Röthlisberger et al., 2017).

**FIGURE 9:**
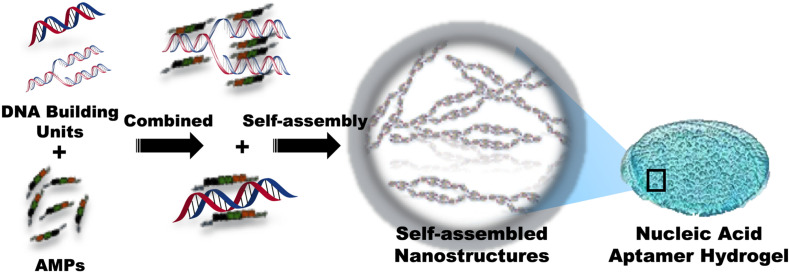
Schematic representation of the composition of nucleic acid aptamer hydrogel with conjugated AMPs.

Over decades, it has been discovered that DNA molecules form multidimensional nanostructures through self-assembly, and connect with various biomolecules to form 2D and 3D structures (Fu et al., 2019). AMP-L12 (LKKLLKKLLKKL) combined with polyanionic DNA nanostructures can form a cross-linked DNA nanostructure hydrogel (Figure 9), with significant bacteriostatic activity against *S. aureus* and MRSA and nuclease-sensitive degradability to control drug release effect. On the other hand, the cross-linked DNA nanostructures have strong anti-inflammatory effects, which can accelerate wound healing (Obuobi et al., 2019). Overall, the cross-linked DNA nanostructures reported in this study might be novel functional wound dressings, with promising prospects for delivering antimicrobial peptides. However, only a few aptamer-functionalized systems have been successfully used in clinical and industrial applications (Ding et al., 2017).

## Self-Assembled Antimicrobial Peptides

Molecular self-assembly refers to the spontaneous formation of well-ordered structures from less ordered states (solution, unfolded conformations, disordered aggregate, etc.), which are driven by thermodynamic and kinetic conditions as a consequence of mutual non-covalent interactions of molecules themselves (Whitesides et al., 2005; Yan et al., 2010; Lombardi et al., 2019a). After undergoing mutual non-covalent interactions, including hydrogen bonds, electrostatic interactions, aromatic stacking, hydrophobic forces, and Van der Waals forces, molecules usually form hierarchal structures with minimal energy state (Han et al., 2010; Yan et al., 2010). Although these non-covalent and individual forces are relatively weak (2–250 kJ mol^–1^), when combined, they can generate highly stable self-association and tune the morphology and function of the assemblies (Lombardi et al., 2019a).

**FIGURE 10:**
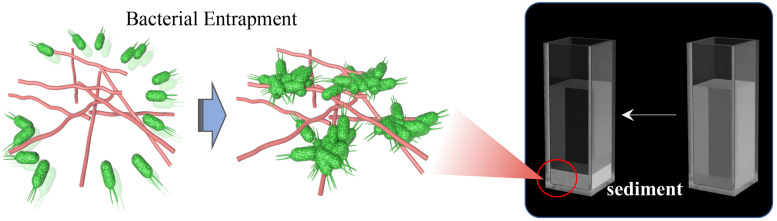
The phenomenon of bacterial entrapment caused by AMP-SAP *in vitro* (Li et al., 2021). Copyright 2021 by the BioMed Central.

Self-assembly occurs spontaneously and ubiquitously in nature, which is critical for vital movement. Examples are represented by DNA double-helix or biological membranes (Mouritsen, 1998; Douglas et al., 2009) and proteins that fold into secondary and tertiary structures (Lentz et al., 2000). Meanwhile, recent studies on innate immunity have also found that some peptides could form ordered nanostructures to interact with bacterial membranes or entrap bacteria to form sediment (Figure 10) (Yeaman and Yount, 2003; Chairatana and Nolan, 2017; Li et al., 2021). Wehkamp J. et al. (Chu et al., 2012; Schroeder et al., 2015; Chairatana et al., 2016) reported that human α-defensin 6 (HD6), a 32-aa cysteine-rich peptide, is stored in Paneth cells (specialized secretory epithelial cells) granules as an 81-residue pro-peptide (proHD6) (Bevins, 2013). When the intestinal mucous tissues were invaded and stimulated by gastrointestinal pathogens (*Listeria monocytogenes* and *Salmonella typhimurium*), the proHD6 was secreted from Paneth cells and further digested by trypsin at the colon lumen, forming the mature activated HD6. These data confirmed that in addition to killing specific microbes in the intestinal environment, HD6 could contribute to self-assembly to form higher-order oligomers termed “nanonet” that binds the protein receptors of the bacterial membrane, entraps intestinal pathogens (*L. monocytogenes* and *S. typhimurium*), and efficiently prevents intestinal pathogen invasion at the mucosal site. Upon aggregation, the HD6–pathogen complex subsequently convenes other immune cells, such as recruited neutrophils, for clearance or direct excretion, thereby triggering a synergistic effect to prevent bacterial resistance. Mechanistically, Phoom C. et al. (Chairatana and Nolan, 2014) described that the hydrophobic index is pivotal in inducing HD6 self-assembly to form the nanonet structure, especially the F2A and F29A mutants of HD6. The replacement of phenylalanine of the 2nd and 29th sites by alanine damaged the transformation of its nanofiber structure so that HD6 failed to aggregate microbes and lost the entrapping action against gastrointestinal pathogens. Thus, the nanonet structure formed by self-assembled HD6 illuminates the ancient and special antimicrobial mechanism of organism immunity systems and simultaneously unmasks the huge application potential of self-assembled functions of AMPs.

In the further insight into self-assemblies of AMPs, in contrast to other building blocks, many investigations discovered that peptide-based supramolecular assemblies have other advantages, including inherent antibacterial activity, preferable biocompatibility, and biodegradability, the simple additional modification of chemistry and biology, the ease of “bottom–up” fabrication, and multiple functions capable of stimuli-responsive recognition (Yan et al., 2010; DeFrates et al., 2018). From an applied point of view, peptides also represent attractive building blocks because of the excellent physical properties such as adhesiveness and flexibility (Unzueta et al., 2015, 2017; López-Laguna et al., 2020). Thus, the review elucidated related literature about the self-assembled AMPs with the improvement of antimicrobial properties. Table 3 summarizes the main representative examples related to self-assembled AMPs described in this section.

**TABLE 3:** Most representative examples of self-assembled AMPs.

**AMP**	**Sequence**	**Self-assembling**	**Antimicrobial activity**	**Tested microorganisms**	**Special functions**	**References**
Diphenylalanine peptide	FF	Aromatic stacking	MIC: 125 μg/ml	*E. coli*	It is the smallest model for antibacterial supramolecular polymers	Schnaider et al., 2017
Fmoc-peptide gelator	Fmoc-FF-Py +	Aromatic stacking	MIC: 10 μg/ml	*S. aureus*	−	Debnath et al., 2010
Fmoc-peptide gelators	NapFFKK	Aromatic stacking	−	*E. coli* and *S. epidermidis*	Reduces the viable *S. epidermidis* biofilm by 94%	Laverty et al., 2014
Tripeptide	^*D*^LFF	Aromatic stacking	−	*E. coli*, *Klebsiella pneumoniae*, and *S. aureus*	Shows the antibiotic delivery capacity, has a mild antimicrobial activity against the Gram-negative strains	Marchesan et al., 2013
HHC-10	KRWWKWIRW-NH_2_	Aromatic stacking	MIC: 8 μM/ml	*S. aureus*	Causes aggregation of bacteria at higher concentrations	Bagheri et al., 2020
A_9_K	AAAAAAAAAK	Hydrophobic forces	A_9_K with concentration of 0.1 mg/ml inhibits the bacterial growth after an incubation of 1 h (survival rate < 20%)	*E. coli* and *S. aureus*	−	Chen C. et al., 2010
RA_9_R	RAAAAAAAAAR	Hydrophobic forces	The concentration to 0.1 wt% RA_9_R reduces the CFU with 2.6, 3.4, and 4.0 orders of magnitude for microbes tested, respectively, after 24 h incubation	*S. aureus*, *P. aeruginosa*, and *P. syringae*	−	Edwards-Gayle et al., 2019
RADA16-Tet213	RADARADARA DARADA-KRWWKWWRRC	Electrostatic interactions and hydrophobic forces	RADA16-Tet213 with concentration of 1 mg/ml eliminates the bacterial cells after 12 h incubation (OD_600_ < 0.3)	*S. aureus*	Has been applied for controlled release delivery of AMPs, promotes the proliferation of bone mesenchymal stem cells	Yang et al., 2018
KLD-3R	RRRKLDL KLDLKLDL	Electrostatic interactions and hydrophobic forces	MIC: 5—6 μM/ml	*E. coli*, *S. aureus*, *B. subtilis*, *P. aeruginosa*	Improves osteogenic property and enhances bone regeneration *in vivo* in a fracture model.	Tripathi et al., 2015
K3W (QL)6K2	KKKWQLQLQ LQLQLQLKK	Intermolecular hydrogen bonding and hydrophobic forces	MIC: 5—20 μM/ml	*E. coli*, *P. aeruginosa*, *S. aureus*, *and S. epidermidis*	Reduces the cytotoxicity on mouse bone marrow-derived monocytes, improves the stability in trypsin and chymotrypsin solutions	Lam et al., 2016
Octapeptide	IKFQFHFD	Intermolecular hydrogen bonding, electrostatic interactions,	MIC: 3 mg/ml at pH 5.5	*S. aureus*	Shows the multiple drug	Wang et al., 2019a
		hydrophobic forces, and aromatic stacking			delivery capacity, exhibits acidic pH-switchable broad-spectrum antimicrobial effect via a mechanism involving cell wall and membrane disruption	
P11-28/29	QQRFEWEFEQQ-NH_2__/_/OQOFO WOFOQO-NH_2_	Intermolecular hydrogen bonding, electrostatic interactions, and aromatic stacking	P11-28/29 with concentration of 10 mg/ml inhibits the bacterial growth after an incubation of 48 h (bacterial growth < 50%)	*P. gingivalis*, *S. sanguinis*	Shows the antibiotic delivery capacity, enables to strengthen the osteogenic differentiation of human dental follicle stem cells	Koch et al., 2019
Human α-defensin 6 (H25W)	AFTCHCRRS CYSTEYSYGTCT VMGINWRFCCL	−	MIC: 32 μg/ml	*Bifidobacterium adolescentis*	Entraps microbes and prevent invasive gastrointestinal pathogens such as *Salmonella enterica serovar Typhimurium* and *Listeria monocytogenes* from entering host cells	Chu et al., 2012; Schroeder et al., 2015

### Diphenylalanine (FF)

As the core recognition motif of the β-amyloid polypeptide, diphenylalanine nano-assemblies are of great clinical interest. Meanwhile, it was identified that diphenylalanine is the most minimalistic nature motif of the peptide-based antimicrobial building block to this day. The pioneering work of Schnaider et al. (2017) demonstrated that diphenylalanine formed unbranched nanotubes while inhibiting the growth of *E. coli* at 125 μg/ml, triggering the upregulation of stress-response regulons, damaging bacterial morphology and resulting in membrane disturbance. Furthermore, tissue scaffolds with antimicrobial capabilities are successfully generated by the incorporation of diphenylalanine into biocompatible matrices. Still, the antimicrobial activity of diphenylalanine is relatively weak. Appropriate modifications should be carried out to develop more effective diphenylalanine-based antimicrobial agents. Such modifications may include the incorporation of high aromatic moieties, such as 9-fluorenylmethoxycarbonyl (Fmoc) or naphthalene (Nap). The increase in aromaticity enhances structural integrity due to the excellent self-assembling properties. Debnath et al. (2010) reported that Fmoc-protected molecules featuring a C-terminal pyridinium (Py^+^) moiety self-assembled into hydrogels (fibers). They displayed moderate antimicrobial activity against both Gram-positive and Gram-negative bacteria. Among all the Fmoc-based amphiphiles, Compound 2 (Fmoc-F-Py^+^) exhibited the most potent antimicrobial activity within 20–50 μg/ml, regardless of the nature of bacteria. However, the inhibitory activity of Compound 4 (Fmoc-FF-Py^+^) against *E. coli* (150 μg/ml) was similar to that of diphenylalanine (Phe–Phe, 125 μg/ml). Also, amphiphile 4 (Fmoc-FF-Py^+^) displayed the lowest MICs (10 μg/ml) against *S. aureus* (Debnath et al., 2010). When it comes to naphthyl-capped cationic peptides, as anti-biofilm coatings for medical devices, NapFFKK hydrogels prominently eradicated the viable *S. epidermidis* biofilm by 94% (Laverty et al., 2014). In addition to the incorporation of high aromatic moieties, modification of terminal functional groups to amino (-NH_2_), the carboxylic acid (-COOH) was also carried out. Peptide nanotubes (NH_2_-FF-COOH) exhibited slightly cell toxicity and effective antimicrobial activity against both planktonic bacteria and biofilm, which eliminated total biofilm at 10 mg/ml against *S. aureus* (3 log_10_ CFU/ml). Unfortunately, NH_2_-FF-COOH was lacking against mutual biofilms of *E. coli* (Porter et al., 2018).

### Surfactant-Like Self-Assembled Antimicrobial Peptides (Surfactant-Like Peptides)

In addition to the stacking interaction described above, the amphipathic nature of the molecules is another significant controlling factor for the self-assembling process of AMPs, which is characterized by the distributed manner of hydrophobic and cationic residues (Zhang W. et al., 2020). Furthermore, the availability of the nanostructure depends on the content of hydrophobicity, as the decrease in the hydrophobicity leads to undesirable nanostructure. Therefore, the well-ordered nanostructures can be obtained by tuning the hydrophobic interaction to keep the hydrophilic–lipophilic balance (Löwik et al., 2005). These peptides are named surfactant-like peptides (SLPs) or lipid-like peptides, which possess a hydrophilic head with one or two hydrophilic residues at the C-terminus with positively charged residues (lysine and arginine) or negatively charged residues (aspartic acid and glutamic acid), and a hydrophobic tail composed of consecutive hydrophobic residues at the N-terminus, e.g., alanine, glycine, valine, and leucine (Vauthey et al., 2002; Zhao and Zhang, 2006; Zhang J.H. et al., 2014). In water or aqueous solutions, the amphiphilic properties of SLPs contributed to the formation of distinct nanostructures, such as nanoparticles, nanofibers, nanorods, or nanotubes (Santoso et al., 2003; Hauser and Zhang, 2010; Zhao et al., 2010; Hamley, 2011; Castelletto et al., 2014; Dehsorkhi et al., 2014; Hamley et al., 2016). Meanwhile, the presence of cationic head groups endows SLPs with antimicrobial capabilities. As self-assembly brings about the concentrating distribution of antimicrobial monomer, SLPs are considered potential antimicrobial agents. The general formula of SLPs was X_*m*_Z_*n*_ (X = hydrophobic residue; Z = cationic residue; *m* = 3, 6, or 9; *n* = 1 or 2), which exhibited different antimicrobial activities and nanostructures by tuning the ratio between different blocks. In the A_*m*_K series, the increased length of hydrophobic tail of SLPs is highly consistent with the improved antimicrobial activity and self-assembly propensity. Hence, the size and shape of the aggregation transition are from loose peptide stacks of A_3_K to long nanofibers of A6K to short and narrow nanorods of A_9_K. The antimicrobial activity of A_9_K is more effective against both *E. coli DH5R* and *S. aureus* than A_3_K and A_6_K. Gratifyingly, A_9_K is much more sensitive to DPPG (a mammalian cell membrane model) than DPPC (bacterial membrane model). Thus, although A_9_K has a long hydrophobic tail, A_9_K has no toxicity to hRBCs over the concentration range for effective antimicrobial activity (Chen C. et al., 2010). Similar to A_9_K, A_9_K_2_ pronounces potent antimicrobial activity against Gram-negative and Gram-positive microorganisms and extremely low mammalian cell cytotoxicity. Hence, A_9_K_2_ hydrogel is considered a potential biomedical material due to its potent bactericidal properties and excellent adhesion and spreading capability to mammalian cells. When speaking of the A_*m*_R series, both A_6_R and A_9_R displays multifunctional self-assembly and bioactivity properties (Castelletto et al., 2018). Especially, A_9_R possessed bacterial activity against challenging clinical microorganisms, while the formation of β-sheet fibers promote the formulation of nanostructured soft materials, ranging from hydrogel formation to emulsion stabilization (Castelletto et al., 2019).

Beyond the layout of X_*m*_Z_*n*_, peptide bola-amphiphiles are another class of surfactant-like peptides, which incorporate a hydrophilic group at either end of the relatively long hydrophobic chain (Edwards-Gayle et al., 2019). The double-head of bola amphiphiles leads to the increase in solubility and the possibility of forming obvious external hydrophilic surfaces (Fuhrhop and Wang, 2004). Thus, compared with A_6_R and A_9_R, which can form nanostructures, only RA_9_R self-assembles into ordered nanofibers. Unlike the A_*m*_R series, RA_9_R has little antimicrobial activity and weaker cytocompatibility than RA_6_R and RA_3_R. Different from the A_*m*_R series SLPs, the increased length of hydrophobic tail of bola amphiphiles does not have a positive correlation with the improved antimicrobial activity and self-assembly propensity (Edwards-Gayle et al., 2019).

### Ionic-Complementary Self-Assembled Antimicrobial Peptides

Similarly, in ionic-complementary peptides, there exist electrostatic interactions driven by charged polar residues (lysine, arginine, aspartic acid, and glutamic acid). The electrostatic force is also an important driving force for the self-assembly process. The previous investigation rendered that the peptide-(RADA)_4_ (sequence-RADARADARADA) with an alternant arrangement between arginine and aspartic acid was employed to form a unique interwoven nanofiber morphology, which could carry other cationic AMPs and provide a sustained-release effect (Yang et al., 2018), but the ionic-complementary peptides (RADARADARADA) were inactive and exerted excellent sterilization properties by carrying other drugs. Hence, the development of ionic-complementary self-assembled AMPs with antimicrobial activity is necessary. It had been reported that the KLD-12, a 12-residue self-assembled peptide driven by ionic-complementary and hydrophobic forces, has import antimicrobial properties by adding several arginines on its N-terminus. These results demonstrated that KLD-12 variants exhibited compelling antimicrobial properties without the increase in cytotoxicity against murine 3T3 fibroblasts and human red blood cells. Additionally, KLD-2R and KLD-3R (RRRKLDLKLDLKLDL) adopted a β-sheet secondary structure as the building block of self-assembly, forming lamellar or amyloid-like nanostructure, which significantly accelerated the formation of a mineralized nodule, enhanced the related gene expression of osteogenic, and improved the osteogenic effect. The ionic-complementary AMPs with antibacterial properties offered more clinical function and application values for self-assembled peptides.

### Intermolecular Hydrogen Bonding Self-Assembled Antimicrobial Peptides

The intermolecular hydrogen bonding formed by glutamine (Gln) was identified as the strongest non-covalent interactions of peptide-based supramolecular assemblies. Nowadays, the self-assembled AMPs triggered by intermolecular hydrogen bonding exert antimicrobial activity mainly in two aspects. First, the self-assembled AMPs, as a whole, perform antibacterial properties. It has been documented that the intermolecular hydrogen bonding and hydrophobic interactions among the (QL) repeating units served to convert peptides from monomer to nanofibers and express other biological functions. Chen W. et al. (2019) described that the peptides-(QL)_6_ consisted of six QL units that were designed as the higher-ordered scaffolds and employed to efficiently improve the biocompatibility of AMPs. As an alpha-helical AMP with a typical membrane-disruptive mechanism, melittin has a strong antimicrobial activity but also has severe cytotoxicity against mammalian cells.

Thus, in combination with peptide-(QL)_6_ scaffolds, the conjugation of (QL)_6_ with melittin dramatically reduced the cytotoxicity of mammalian cells by changing the conformation of melittin from helical structure to sheet structure, resulting in the transformation of antimicrobial action of melittin. Further mechanistic analysis indicated that the peptide-(QL)_6_ scaffolds impaired the hydrophobicity of melittin, thereby, reducing the sensitivity and permeability of melittin to cytomembrane lipids, subsequently decreasing the damage to mammalian cells. Following the availability of the improved biocompatibility scaffolds of (QL)_6_, the team also modified the self-assembled AMPs based on the (QL)_6_ scaffold and obtained other functions, including excellent biocompatibility and protease stability. Xu et al. (2015) designed self-assembling antimicrobial nanofibers (SAANs) with a general formulation of K_*x*_W(QL)_*y*_K_*z*_ (K: lysine, Q: glutamine, L: leucine, W: tryptophan), where x, y, z represents the number of the repeating units. One of the peptides, K_3_W (QL)_6_K_2_, facilitated the formation of stable β-sheet nanofibers driven by intermolecular hydrogen bonding and hydrophobic interactions. Meanwhile, the cationic properties of lysine and the hydrophobicity of tryptophan carried excellent and broad antimicrobial activity (MIC from 5–20 μM). In addition to showing satisfactory stability in trypsin and chymotrypsin solutions, these K_*x*_W(QL)_*y*_K_*z*_ formulaic peptides at high concentrations also significantly reduced the cytotoxicity, maintaining approximately 100% cell survival. Mechanistically, the results of NMR spectroscopy revealed that the K_*x*_W(QL)_*y*_K_*z*_ formulaic peptides can form ordered and stable supramolecular β-sheet assembly when contacting with the lipid membrane of microbes. Meanwhile, direct imaging conducted by TEM assay further illuminated that the antimicrobial efficiency of K_*x*_W(QL)_*y*_K_*z*_ formulaic peptides were motivated by the interaction between the nanofiber of AMPs and bacterial membrane, achieving the deformation and disturbance of membrane, causing the death of bacteria (Xu et al., 2018).

**FIGURE 11:**
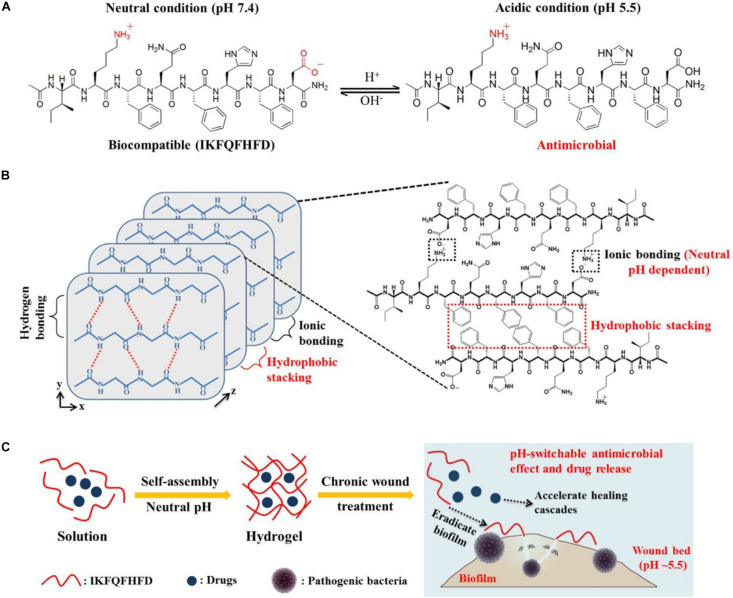
**(A)** Peptide sequence of IKFQFHFD under neutral and acidic conditions. **(B)** Principle of IKFQFHFD to form self-assembled nanofiber networks hydrogel at neutral pH. **(C)** Conceptual illustration of self-assembled nanofiber networks hydrogel for biofilm eradication and therapy method in a chronic wound (Wang et al., 2019a). Copyright 2019, American Chemical Society.

Second, AMPs utilized the self-assembling capacity to form nanofiber networks via intermolecular hydrogen bonding and other multiple forces (electrostatic interactions, aromatic stacking, hydrophobic forces) so that they can encapsulate other antimicrobial agents and possess certain antimicrobial activity. Franziska K. et al. (Koch et al., 2018, 2019) aimed to search for suitable candidate materials for periodontal therapy. It was found that the self-assembled peptide P11-28/29 (QQEFEWEFEQQ-NH_2_/OQOFOWOFOQO-NH_2_) contributed to the formation of complicated nanofiber hydrogels due to the interaction of multiple forces (intermolecular hydrogen, electrostatic interactions, aromatic stacking). Results showed that P11-28/29 had a significant antimicrobial effect against the periodontal pathogen *Porphyromonas gingivalis* and strengthened the osteogenic differentiation of human dental follicle stem cells simultaneously. Furthermore, the P11-28/29 was employed to exert excellent cargo-loading capacity and sustained release function. It can encapsulate these antibiotics stably and exhibit a sustained-release effect on a lesion over 120 h, demonstrating the compelling application advantage of the P11-28/29. Similarly, to efficiently kill bacteria, eradicate biofilm, and cure stalled healing in a chronic wound, Wang et al. (2019a) designed a self-assembled peptide octapeptide (IKFQFHFD) containing all non-covalent interactions to form 3D nanofiber networks of hydrogel to ensure stable encapsulation of other agents (photothermal agent and procollagen component) (Figure 11). After that, the insertion of histidine in a peptide sequence endowed octapeptide the pH-modulating ability, which can self-assemble at neutral pH and dissociate into active free monomer at pH 5.5. The results *in vitro* rendered that the octapeptide expressed a stable supramolecular nanofiber network structure and maintained excellent drug encapsulation efficacy at neutral pH. When the octapeptide encountered the infectious wound at acidic pH (environment of infected chronic wounds), it displays an appealing antimicrobial effect through the release of free octapeptide and the exhibition of cationic properties under acidic condition. In addition, the photothermal agent would be released in the lesion, causing the destruction of biofilm on the wound surface via NIR laser irradiation. Meanwhile, the results in the epidermal wound model showed that the conjugation of octapeptide with the photothermal agent and procollagen component not only significantly decreased the number of bacterial colonies but also induced subsequent healing cascades and activated cell proliferation simultaneously, including the shortening of inflammation, the acceleration of surface collagen deposition, revascularization, and scar formation.

Thus, structurally speaking, different non-covalent bonds can form various nanostructures, such as fibers, tubes, tapes, micelles, or network nanostructures. Meanwhile, the different nanostructures also are changed with different concentrations of the same peptide sequence. Additionally, functionally speaking, self-assembled peptides mainly had two patterns to solve the infectious issue. One is the self-assembled peptides with antimicrobial properties, the other is the self-assembled peptides with the encapsulation and controlled drug release functions. These abundant nanostructures and functions of self-assembled peptides play a pivotal role in many biomedical applications, particularly in drug delivery, wound healing, and tissue engineering.

## The Impact of Antimicrobial Properties With Nanosystems Containing Antimicrobial Peptides

With the gradual understanding of the structure–function relationship of AMPs on the molecular level, AMPs have become the first choice for the substitution of antibiotics (Nagarajan et al., 2019). However, AMPs still have some disadvantages that seriously limit their development, including the fragile internal half-life, high susceptibility to proteolytic enzyme, and cytotoxicity of eukaryote (Wang et al., 2019c; Shao et al., 2021). The emergence of Ns-AMPs has important implications for medical treatment application. It has been documented that Ns-AMPs, in addition to promoting the intrinsic antimicrobial activity and stability, and decreasing the cytotoxicity, also has the function of site-directed targeting and controlled drug release to remedy the defects of monomeric AMPs (Lombardi et al., 2019a; Lopez-Silva et al., 2019; Wang et al., 2019a). However, the actual situation is that it is not generalized for all of Ns-AMPs to improve their intrinsic antimicrobial properties or obtain the abovementioned special functions because the Ns-AMPs formed by different material types, and binding ways have different antimicrobial properties and biological functions (Martin-Serrano et al., 2019; Carratalá et al., 2020; Thapa et al., 2020). A deeper understanding of the physical and chemical parameters of Ns-AMPs is helpful to display its advantages of antimicrobial properties well and make up for its shortcomings. Thus, the antimicrobial properties of Ns-AMPs, such as antimicrobial activity, cytotoxicity, stability, and controlled-release function, are summarized.

In contrast to other Ns-AMPs, the conjugation of MNPs with AMPs facilitates the enrichment of AMPs on the MNP surface, which enables AMPs to form encapsulating matrix of MNPs to improve integral antimicrobial properties. It has been reported that the AMP–MNP conjugate has an excellent antibacterial activity (Gao et al., 2020; Jin et al., 2021). The main reasons are the following: (1) Both AMPs and MNPs (mainly AgNPs and AuNPs) display potent antimicrobial activity, which is the basis for maintaining the antimicrobial activity of the AMP–MNP conjugates. (2) The enrichment of AMPs on MNP surface contributes to the increase in local AMP concentration, increasing the contact area with the bacterial membrane (Peng et al., 2016). (3) AMPs and MNPs have a potentially antibacterial synergistic effect against microbes due to their special antimicrobial mechanism (Zheng et al., 2019; León-Buitimea et al., 2020). Some literature showed that both AgNP–AMP and AuNP–AMP conjugates served to damage the bacterial outer membrane and disturb the permeability of the bacterial membrane, causing the leakage of intracellular content (Mei et al., 2013; Lee et al., 2017; Zheng et al., 2019; Gao et al., 2020). Once there is entry into a cell, Au/AgNPs could evoke the killing pathway of nitric oxide-independent manner, continuously motivating the fragmented destruction of bacterial DNA (Mohanty et al., 2013; Zheng et al., 2019), finally resulting in the death of microbes. Meanwhile, the MNP–AMP is less prone to develop drug resistance due to their multiple antimicrobial actions and the damage to bacterial DNA (Slavin et al., 2017). Additionally, because certain MNPs (AgNPs) possess severe toxicity *in vivo*, another vital reason for the combination of MNPs with AMPs is that AgNPs internalized by the bundling of AMPs decrease the binding effect on eukaryote and weaken their intrinsic cytotoxicity (Makowski et al., 2019; D’Souza et al., 2020; Falanga et al., 2020). However, some studies described that the protease stability of MNP–AMP is partly improved but is invalid in the presence of high concentration protease (Casciaro et al., 2017) because the MNP–AMP promotes the aggregation of AMPs on the surface of MNPs and further forms a stable nanostructure containing external AMP bundling and internal MNPs. Thus, the decorated nanostructure contributes to the formation of the close-packed array of AMPs and slightly decreases the combination of AMPs with protease. Still, the accumulation of AMPs on the external of the decorated nanostructure maintains the strong binding force between protease and the bundling of AMPs, causing the lower anti-protease efficiency, compared with other N-AMP.

The CNTs are considered the cutting-edge biological materials because they provide plentiful binding sites of drugs and improve the dispersibility and biocompatibility for other drugs (Bianco et al., 2005). However, the CNTs only help the AMPs improve the biocompatibility of mammalian cells and had little effect on the enhancement of antimicrobial capacity (Xu M. et al., 2020). Meanwhile, the expensive synthesis of CNTs, the definition of class 2B carcinogens, and the poor knowledge systems of the establishment of CNTs also lead to the slow progress on biomedicine.

Thus, the combination of inorganics with AMPs provides great value for the development of antibacterial nanomaterials, especially for the development of antimicrobial medical equipment. However, long-term toxicity assay *in vivo* should be conducted because the conjunction of inorganics with AMPs still expresses cytotoxicity, inflammatory response, and immune response even *in vitro*. Moreover, the influences of inorganic materials deposited in the human kidneys also needed to be investigated.

Similar to other Ns–AMPs, the organic material nanosystems containing AMPs could be categorized into organic material nanosystems with conjugated AMPs and organic material nanosystems with encapsulated AMPs, but the diversity of a combination of ways and material species of organic materials also has an effect on the antimicrobial activity, biocompatibility, and stability of AMPs. Overall, the organic material nanosystems, which can combine with AMPs and form a hydrophilic shell to internalize AMPs, or that can encapsulate AMPs and achieve controlled release of AMPs, generally weaken antibacterial activity (Kumar et al., 2015), or partially maintain the sterilization effect (Jiang et al., 2018), including polymers such as PEG (Cleophas et al., 2014; Gong et al., 2017), PLGA (Vijayan et al., 2019), chitosan (Qi et al., 2017; Sun T. et al., 2018), hyaluronic acid (Kłodzińska et al., 2019), liposomes (Boge et al., 2016, 2017, 2019; Håkansson et al., 2019), nucleic acids (Fu et al., 2019; Obuobi et al., 2019), and cyclodextrins (Cruz Olivo et al., 2017; Zhang et al., 2018). The reason is that the AMPs encapsulated in organic material nanostructure are difficult to contact with pathogens, leading to the loss of antimicrobial activity (Kumar et al., 2015), while the AMPs loaded on the surface of organic material nanostructure generally maintain the intrinsic antimicrobial effect (Jiang et al., 2018). However, the disappointing antibacterial capacity of AMP-containing organic material nanosystems does not need to be of excessive concern due to the appearance of novel abilities. Many organic materials can be used to construct a targeted and controlled drug release system that adjusts the release of AMPs, increases the drug concentration at local sites (Qi et al., 2017), strengthens the antimicrobial ability at specific sites (Sun T. et al., 2018; Falciani et al., 2020; Ghaeini-Hesaroeiye et al., 2020; Feng et al., 2021), and reduces the overall drug circulation, cytotoxicity, and side effects (Akbarzadeh et al., 2013; Almaaytah et al., 2017; Mao et al., 2017; Kłodzińska et al., 2019; Sanches et al., 2021). In addition, the encapsulation can act as a barrier to protect the AMPs from the recognition of the immune system, the clearance of the reticuloendothelial system, and even the degradation of proteases, ultimately improving the half-life of the AMPs *in vivo* (Qi et al., 2017; Obuobi et al., 2019). Meanwhile, the combination of organic material with AMPs increases the apparent integral size, reducing the renal filtration capacity and altering the biological distribution (Qi et al., 2017). These advantages significantly improve not only the cell selectivity and anti-protease stability of AMPs but also truly improve the antimicrobial properties *in vivo* simultaneously. Therefore, polymeric materials with antibacterial ability have promising prospects as auxiliary medical materials, but the biosecurity issues and residual problems *in vivo* are still to be studied.

**FIGURE 12:**
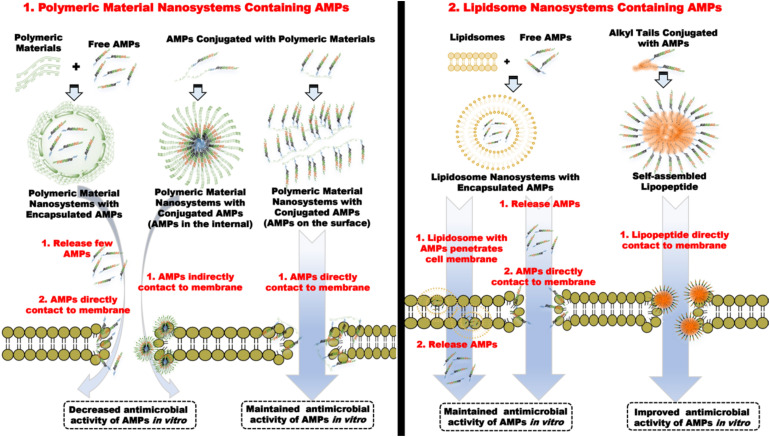
The typical antimicrobial mechanisms of most organic material nanosystems containing AMPs.

Additionally, some organic–AMP nanosystems can enhance the antimicrobial activity of AMPs, as well as lipopeptides and liposome-encapsulated nanosystems. Some AMPs without membrane-destructive bactericidal mechanism are encapsulated within liposomes to form spherical nanosystems. Because liposomes have a good affinity for bacterial membranes, liposome nanosystems can carry AMPs into the cells of pathogens. The encapsulated AMPs are released after entering the cell and targeted to a specific intracellular site to achieve the elimination of bacteria (Cantor et al., 2019; Sanches et al., 2021). However, for other membrane-active AMPs, liposome nanosystems failed to improve their antimicrobial activity (Håkansson et al., 2019). In general, the lipopeptides have been pointed out as the self-assembled AMPs. Most lipopeptides have strong antimicrobial properties because the fatty acids, as the hydrophobic tail of AMPs, supply sufficient hydrophobicity for AMPs to improve antimicrobial activity (He et al., 2018; Lei et al., 2018; Stachurski et al., 2019) and form a self-assembled nanostructure, thereby, improving the stability of salt and protease resistance (Immordino et al., 2006), and achieving the promotion of overall antimicrobial properties. Figure 12 introduces the universal antimicrobial models for most organic material nanosystems containing AMPs. Unfortunately, it is a fatal defect that lipopeptides cause serious hemolysis and cytotoxicity of erythrocytes (de Almeida et al., 2019; Lombardi et al., 2019b). Thus, how to solve the toxicity issue of lipopeptides has become a crucial factor in clinical application.

**FIGURE 13:**
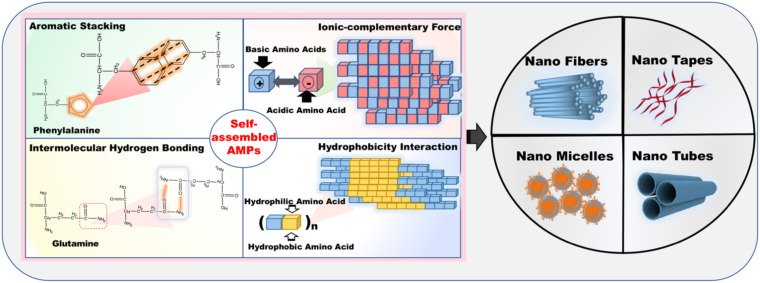
The various acting forces (aromatic stacking, ionic-complementary force, intermolecular hydrogen bonding, and hydrophobicity interaction) of self-assembled AMPs and the self-assembled AMPs formed by these representative amino acids.

Free AMPs utilize the non-covalent interactions, including intermolecular hydrogen bonds, electrostatic interactions, aromatic stacking, and hydrophobic forces to form various nanostructures (Figure 13). Mechanically, self-assembled AMPs exert antimicrobial activity mainly through the continuous release of monomer peptides from a specific environment or keeping an intact nanostructure (Lombardi et al., 2019a; Wang et al., 2019a). The vital factors for the antimicrobial activity of monomer AMPs are hydrophobicity, positive charge, and amphipathicity, and the same is true of self-assembled AMPs (Liu et al., 2013; Schnaider et al., 2017). Generally, the antimicrobial activity of most self-assembled AMPs was weaker than that of monomer peptides, especially for the self-assembled AMPs bound by intermolecular hydrogen bonding and complementary binding of charged amino acids, but the self-assembled AMPs formed by hydrophobic interaction or aromatic stacking have a better antimicrobial activity than those formed by other binding forces (Yang et al., 2018; Chen W. et al., 2019). The reasons are as follows: First, glutamine (Gln) is the typical amino acid that is capable of forming intermolecular hydrogen bonds and usually destroys the amphiphilicity of the AMPs. Second, ionic-complementary peptides lose their cationic due to the neutralization of positive and negative charged amino acids. Third, the self-assembled AMPs formed by hydrophobic interaction or aromatic stacking maintain the higher hydrophobicity (Chou et al., 2016; Buommino et al., 2019).

In contrast to free AMPs, although self-assembled AMPs are less effective in inhibiting the microbes, self-assembled AMPs formed by a variety of covalent bonds are still able to significantly decrease the cytotoxicity to mammalian cells (Xu et al., 2018; Chen W. et al., 2019). That is still closely related to the forces formed by the self-assembled AMPs. In general, the self-assembled AMPs formed by ionic-complementary or intermolecular hydrogen bonds have excellent biocompatibility because their cell membrane permeability is impaired by Gln or negatively charged amino acids. Moreover, due to the interaction between aromatic amino acids or hydrophobic amino acids, the self-assembled AMPs formed by hydrophobic interaction or aromatic stacking hinder the combination of peptides and bacterial membrane to a certain extent, thereby improving the cell selectivity, but there are exceptions. For example, self-assembled AMPs of RA_9_R produces strong cytotoxicity, probably due to the high hydrophobicity of RA_9_R (Chen C. et al., 2010; Castelletto et al., 2018). Hence, in general, the increase in cell selectivity and the decrease in antimicrobial activity in most self-assembled AMPs are complementary.

Although self-assembled AMPs have such defects *in vitro*, different types and functions of hydrogels are formed through different non-covalent bonding forces, which makes AMPs obtain more attractive application prospect. For example, self-assembled AMPs formed by intermolecular hydrogen bonding and ionic-complementary significantly reduce the cytotoxicity and exhibit a sustained drug release profile (Zhang et al., 2005). Except for the controlled release function, self-assembled AMPs formed by hydrophobic force and aromatic stacking also display a nice activity against the pathogen (Marchesan et al., 2013). Therefore, some self-assembled AMPs form nanosystems based upon the interactions of multiple covalent bonding forces so as to obtain hydrogel scaffold materials with low cytotoxicity that can encapsulate other drugs and have a sustained-release effect (Koch et al., 2019; Wang et al., 2019a). Hydrogel scaffold materials have some advantages. First, the complexed “nanofiber network” structure can form hydrogels, which endure AMPs with the capability to carry a variety of drugs and obtain sustained-release effect. Second, the formed self-assembled AMPs can significantly increase the size of AMPs, reduce the clearance of the reticuloendothelial system and the recognition of the immune system, prolong the half-life of drugs *in vivo*, and improve the stability of the AMPs (Jeong et al., 2013; Mangelschots et al., 2016). Third, by adding a specific amino acid (histidine), controlled release nanosystems with specific environmental response can be obtained, which not only reduces the side effects *in vivo* but also increases the drug concentration at local sites to make up for the reduced antimicrobial activity of self-assembled AMPs (Guo et al., 2010; Lu et al., 2015). It can be said that the above advantages are crucial for the further application of AMPs *in vivo*. On the basis of the above studies, it will become a director with great research potential to explore the synergistic effect of self-assembled AMPs and encapsulated drugs (e.g., antibiotics and AMPs) in the future.

The half-life stability of self-assembled AMPs is indeed improved *in vivo* due to the formation of the large-size nanostructure. Moreover, its anti-protease stability has also been improved because the more close-knit structure formed by self-assembly of AMPs efficiently hinders the combination of restriction site to proteases, but self-assembled AMPs always belong to small-molecule peptides, regardless of how stable the self-assembled nanostructures are. Since the stability of self-assembled AMPs is weaker than that of organic material nanosystems, a high concentration of protease can completely cleave the sequence of self-assembled AMPs, resulting in the loss of the antimicrobial ability of AMPs (Chen and Liang, 2013; Xu et al., 2015). Thus, the self-assembled AMPs as the antimicrobials agents *in vivo* have limitations in the clinical application, and the anti-protease stability still needs to be further explored. Nowadays, self-assembled AMPs attracted more attention in the field of medical antibacterial materials, wound healing scaffolds, and controlled drug release frameworks.

## Conclusion and Perspectives

Intensive efforts have been devoted to developing various nanosystems containing AMPs to deal with the defects of free AMPs, such as high cytotoxicity, poor pharmacokinetic profiles, finally handling the unprecedented threats from multidrug-resistant pathogens. In this context, we summarized the recent discoveries in the development of the nanosystem containing AMPs, e.g., inorganic material nanosystems with conjugated AMPs, organic material nanosystems containing AMPs and self-assembled AMPs. Universally, the Ns-AMPs, especially polymeric and liposome nanosystems containing AMPs, improved the internal half-life and the pharmacokinetic profiles *in vivo* due to their large-size nanostructure, while acquiring the drug targeting and controlled-release function that enables them to carry other drugs to assist antimicrobial treatment. Additionally, the lipopeptide and the MNPs with conjugated AMPs generally exhibited excellent antimicrobial activity and dramatically antimicrobial synergistic effect. Based on the flexibility of peptide sequence and no residue *in vivo*, self-assembled AMPs, in addition to enhancing the biocompatibility of mammalian cells, were utilized as the drug-targeting material, which conducted the responsive release of AMPs in the microenvironment of bacterial infection sites (e.g., acidity or bacterial enzymes). Thus, the applications of N-AMP are promising to efficiently solve the emergence of existing antibiotic resistance and quickly realize the transition of applications of AMPs from *in vitro* to *in vivo*.

In the future, many crucial clinical issues are still standing in front of researchers. For example, although various nanostructures (tubes, fibers, tapes, or micelles) with different functions had been designed, the formation of these nanostructures is still hardly regulated and forecasted. Different types of AMPs and conjugated materials had a huge impact on nanostructures. Thus, the Ns–AMPs studies on structure–function relationship needed to be deeper explored. Additionally, the stability studies of Ns–AMPs still overstayed *in vitro*; the relevant assays *in vivo* needed to be performed, especially more intuitive fluorescence imaging and sufficient results of half-life kinetics in representative animal models (e.g., mice or swine). Then, the long-term toxicity of Ns–AMPs in the body should be further investigated due to the existence of non-degradable polymeric materials. Additionally, some nanosystems containing AMPs with the synergistic antimicrobial effect can be considered as tremendous research potential.

## Author Contributions

ZY, SH, and AS conceptualized the study. ZY, SH, HW, and TY prepared and wrote the original draft. ZY and LW handled the graphic design. AS was in charge of the supervision. All authors contributed to the article and approved the submitted version.

## Conflict of Interest

The authors declare that the research was conducted in the absence of any commercial or financial relationships that could be construed as a potential conflict of interest.

## Publisher’s Note

All claims expressed in this article are solely those of the authors and do not necessarily represent those of their affiliated organizations, or those of the publisher, the editors and the reviewers. Any product that may be evaluated in this article, or claim that may be made by its manufacturer, is not guaranteed or endorsed by the publisher.
